# Mustache: multi-scale detection of chromatin loops from Hi-C and Micro-C maps using scale-space representation

**DOI:** 10.1186/s13059-020-02167-0

**Published:** 2020-09-30

**Authors:** Abbas Roayaei Ardakany, Halil Tuvan Gezer, Stefano Lonardi, Ferhat Ay

**Affiliations:** 1 Centers for Autoimmunity and Cancer Immunotherapy, La Jolla Institute for Immunology, La Jolla, 92037 CA USA; 2 Computer Science and Engineering, University of California, Riverside, Riverside, 92521 CA USA; 3 Computer Science and Engineering, Sabanci University, Tuzla, Istanbul, 34956 Turkey; 4 School of Medicine, University of California, San Diego, San Diego, 92093 CA USA

**Keywords:** Contact maps, Genome architecture, Chromatin loops, Hi-C, Micro-C, HiChIP, ChIA-PET, Promoter-enhancer interactions, CTCF, Cohesin

## Abstract

We present Mustache, a new method for multi-scale detection of chromatin loops from Hi-C and Micro-C contact maps. Mustache employs scale-space theory, a technical advance in computer vision, to detect blob-shaped objects in contact maps. Mustache is scalable to kilobase-resolution maps and reports loops that are highly consistent between replicates and between Hi-C and Micro-C datasets. Compared to other loop callers, such as HiCCUPS and SIP, Mustache recovers a higher number of published ChIA-PET and HiChIP loops as well as loops linking promoters to regulatory elements. Overall, Mustache enables an efficient and comprehensive analysis of chromatin loops. Available at: https://github.com/ay-lab/mustache.

## Background

Recent studies have revealed that chromatin has a well-organized structure in the eukaryotic nucleus, which is highly regulated in accordance with the stage of the cell cycle, environmental cues, and disease conditions [1–4]. In turn, the 3D structure of the chromatin plays a critical role in many essential cellular processes, including the regulation of gene expression and DNA replication [5–7]. Therefore, it is of great importance to systematically study the chromatin organization to understand how folding properties and looping events influence the cell-specific biological functions of distinct regulatory regions of the DNA. To date, Hi-C has been the main assay of choice for discovering genome-wide chromatin contacts/interactions [8, 9]. More recently, Micro-C [10], which replaces the restriction enzyme in Hi-C with micrococcal nuclease for digestion, enabled the generation of nucleosome-resolution chromosome folding maps from mouse and human cells [11, 12]. With the decreasing cost of sequencing and optimization for smaller cell numbers, both Hi-C and Micro-C assays are expected to produce increasingly higher resolution reference contact maps for a diverse set of organisms and cell types [9, 12, 13].

Chromatin loops are defined as pairs of genomic sites that lie far apart along the linear genome but are brought into spatial proximity by a mechanism called *loop extrusion* [14–16]. Several methods have been developed to detect chromatin loops or statistically significant/enriched chromatin interactions from Hi-C contact maps [9, 17–20]. These existing methods broadly fall into two groups. The first group, which we call *global enrichment-based methods*, contains methods that (i) globally fit statistical/probabilistic models to the contact map data and (ii) assign *p* values to each individual pixel/entry in the contact map by comparing the observed count values to the expected values computed from the fitted model. For instance, Fit-Hi-C [17] uses a monotonic spline to model the relation between contact probability and the genomic distance of the interacting loci, then estimates the statistical confidence of each contact with respect to this expectation and a coverage-based correction factor, using a binomial distribution. In a similar method, HiC-DC [18] estimates the statistical significance of chromatin contacts from Hi-C experiments using a hurdle negative binomial regression to account for both the zero inflation and over-dispersion of contact counts as well as systematic sources of variation in Hi-C read counts such as distance-dependent random polymer ligation, GC content, and mappability bias. Several crucial drawbacks common to these two methods, and to any other method that uses only a global background [20], are as follows: (i) the locality information in the contact map is not taken into account in the modeling, (ii) each pixel/contact count is considered independent of its surrounding pixels, and (iii) pixels that are in the vicinity of a strong loop are also deemed statistically significant with respect to the global background (i.e., bystander effect). As a consequence, a large number of reported significant contacts are likely to cluster around a few very strong pixels or loops, making it difficult to interpret the large number of direct and indirect enrichment lumped together. Recent variations of these methods employ post-filtering strategies for discarding calls that are likely to be indirect interactions [21, 22]; however, for deeply sequenced Hi-C data, the number of resulting calls still remains very large (e.g., in the order of hundreds of thousands). Therefore, the global enrichment-based methods, even though are important for discovering preferential enrichment of proximity among functional regulatory elements such as promoters and enhancers [22], are not for highly specific detection of the strongest structural loops, such as those demarcating domain boundaries and in between convergent CTCF binding sites [9].

The second group of methods, which we name *local enrichment-based (loop calling) methods*, identifies 2D peaks in contact map that are not only significantly higher than expected from the global background, but also are *local maxima* with respect to their neighboring pixels. For example, Rao et al. developed a method called Hi-C Computational Unbiased Peak Search (HiCCUPS) that detects chromatin loops in deeply sequenced high-resolution Hi-C maps [9]. HiCCUPS examines each pixel (locus pair) in the contact map by comparing its contact frequency to that of neighboring pixels. More specifically, HiCCUPS identifies loops by finding “enriched” pixels, that is, locus pairs whose contact counts are significantly higher than that of (1) pixels to its lower-left, (2) pixels to its left and right, (3) pixels above and below, and (4) a doughnut-shaped region surrounding the pixel of interest. While our work was under review, a method called Significant Interaction Peak caller (SIP) was published which also uses image processing techniques to identify chromatin loops in Hi-C data [19]. SIP applies a set of image processing operations (e.g., Gaussian blurring and contrast enhancement) to pre-process the contact maps and increase the contrast of the potential loops. Then, SIP employs a local maxima detection algorithm to produce the preliminary list of candidate loops, which undergo several filtering steps to produce the final set of loop calls. Both HiCCUPS and SIP use a fixed representation of the contact map and a fixed-size local neighborhood to model the background intensities. Therefore, chromatin loops involving proximity of larger (or smaller) regions that lead to larger (or smaller) blobs in the contact map, as compared to the scale of the fixed representation, may not meet the local filtering criteria and will not be reported.

Another recent approach called cLoops is a local-enrichment method based on a modified DBSCAN clustering algorithm (cDBSCAN) that directly works with the paired-end tags/reads (PETs) to call loops [23]. cLoops uses a permuted local background to estimate statistical significance and can handle a broad range of chromatin conformation capture assays including Hi-C, ChIA-PET, HiChIP, and Trac-loop. While cLoops is, to some extent, scale-free since it utilizes reads at their native resolution, cLoops is very inefficient both in terms of runtime (>1000× slower compared to SIP [19]) and memory use (> 100 GB per chromosome). Another downside is that cLoops reports loops that are not supported by either HiCCUPS, SIP, or Mustache, whereas these three methods have strong agreement among each other. For these reasons, we focused on comparing the three local enrichment-based methods, namely Mustache, SIP, and HiCCUPS.

**Fig. 1:**
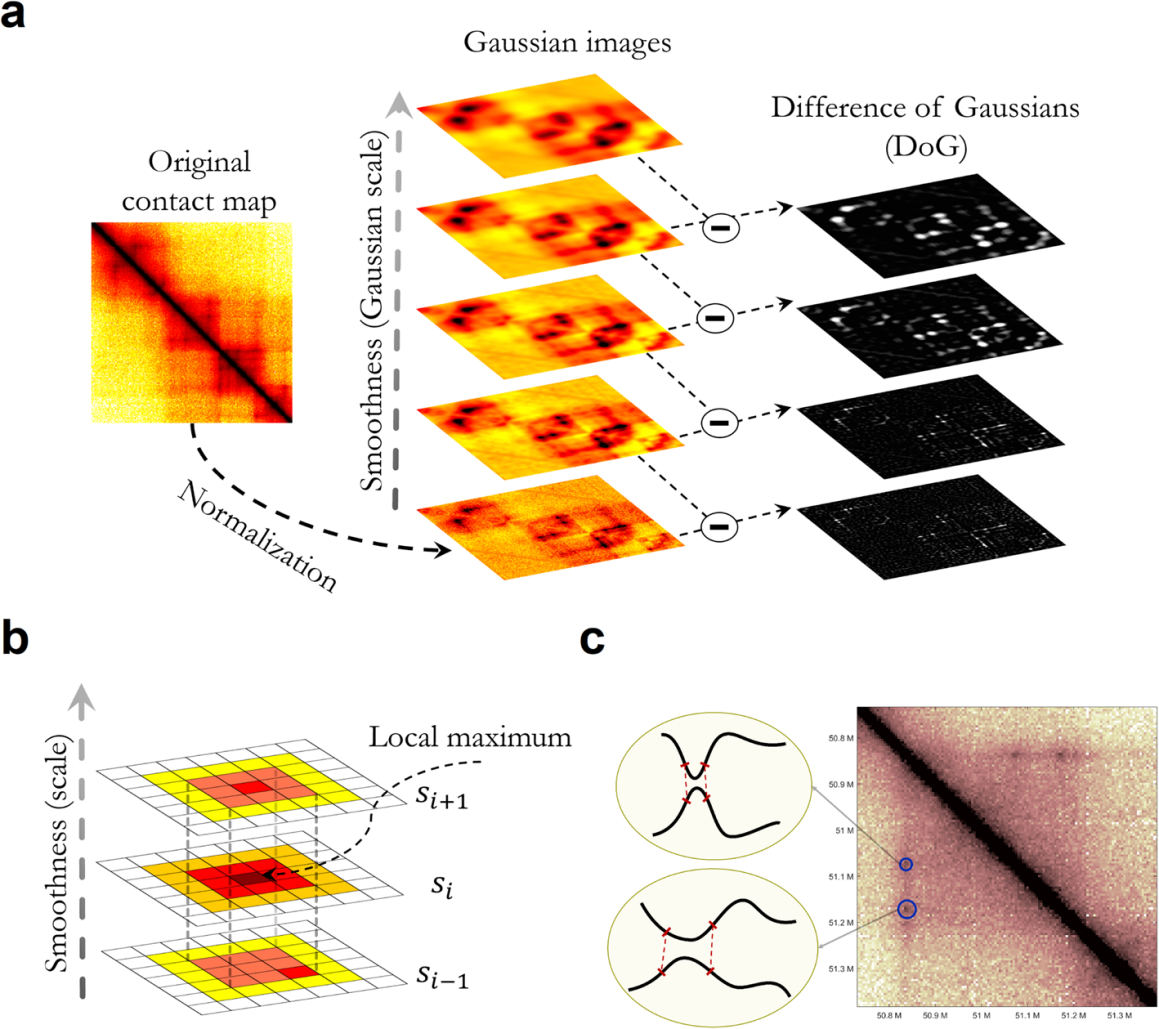
**a** The initial contact map is repeatedly convolved with increasing 2D Gaussians to produce a scale-space representation of the image (shown on the left). Pairwise adjacent Gaussian images are subtracted to produce the difference-of-Gaussian (DoG) images (on the right). **b** Maxima of the difference-of-Gaussian images are detected by comparing each pixel to its 3 ×3×3 neighborhood in (*x*,*y*,*σ*) space. Note that DoG is a local maximum at (*x*,*y*) location at scales *s*_*i*_ and *s*_*i*+1_ but not at scale *s*_*i*−1_, therefore passing the first filtering step criteria. **c** Chromatin loops can be caused by the contact between pairs of DNA segments at different scales

In this paper, we present MUSTACHE, a new local enrichment-based method for high-resolution Hi-C and Micro-C data. MUSTACHE uses the scale-space representation of a contact map to model and identify chromatin loops at multiple resolutions (Fig. 1). MUSTACHE utilizes a set of carefully designed filters to report only locally enriched pixels as loops. Our experimental results show that MUSTACHE detects chromatin loops that are reproducible, have high support from aggregate peak analysis, and are independently supported by other conformation capture experiments as well as by genomic and epigenomic correlates of loop formation. Given the orders of magnitude of difference in the resulting calls from MUSTACHE and global enrichment-based methods such as Fit-Hi-C and HiC-DC (e.g., 20k vs 1.1M and 700k, respectively), and the problematic issues outlined above related to cLoops, here we focus on comparing MUSTACHE to the most commonly used loop calling method HiCCUPS [9] and a very recent and similar image processing approach SIP [19]. Our comparisons show that MUSTACHE provides better statistical power in detecting loops compared to HiCCUPS and SIP while detecting the majority of their reported loops. We present several lines of evidence suggesting that the additional loops detected only by MUSTACHE are not false positives, but are rather *bona fide* looping events with visible enrichments in contact maps and support by other conformation capture assays. We also demonstrate MUSTACHE’s efficiency on Micro-C contact maps leading to highly consistent loop calls between Micro-C and Hi-C maps of the same cell line [12]. Our scalable implementation of MUSTACHE also allowed us to study 1 kb Micro-C maps and to include regions less than 20 kb apart, which have traditionally been challenging to study with Hi-C data and existing loop callers. This high-resolution analysis revealed loops that are highly supported by CTCF binding and APA plots as well as thousands of new loops involving promoter and enhancer regions at a resolution that allows studying such regulatory elements individually. Based on the results presented here, we believe that MUSTACHE will become an essential tool in the analysis of high-resolution Hi-C and Micro-C contact maps, which are being produced in large numbers by the 4D Nucleome project and other efforts [24].

## Results

### Mustache detects chromatin loops from publicly available contact maps

We ran MUSTACHE on (i) Hi-C contact maps for human cell lines GM12878 and K562 obtained from Rao et al. [9], (ii) Hi-C and Micro-C contact maps of HFFc6 cell line obtained from Krietenstein et al. [12], and (iii) Micro-C contact maps of mouse embryonic stem cell (mESC) from Hsieh et al. [25] (Additional file 1: Table S1). For assessing the consistency of loop calls at different resolutions and for measuring reproducibility across replicates, we also used 10 kb GM12878 Hi-C contact maps. All contact maps were produced using a minimum read alignment quality MAPQ ≥ 30. GM12878, K562, and HFFc6 Hi-C data consisted of approximately 4.9B, 1B, and 3B valid read pairs, respectively. HFFc6 and mESC Micro-C data had 4.4B and 2.6B valid read pairs, respectively. For all experiments at 5 kb or 10 kb resolution, we considered contacts within the genomic distance range of 20 kb to 2 Mb. For 1 kb resolution Micro-C analysis, we used 5 kb to 2 Mb distance range. For all cases, we employed MUSTACHE with default parameters: *σ*_0_=1.6,*s*=10 and *oc*=2,*st*=0.88, where *σ*_0_ is the initial scale, *s* denotes the number of scales/levels, *oc* denotes the number of octaves, and *st* refers to the sparsity threshold as described in the “Methods” section. We selected these datasets because they are the most extensively studied, they are generated with very high-depth sequencing, and these cell lines have readily available orthogonal datasets including ChIP-seq, ChIA-PET, and HiChIP, which we used for validating our results. For the 5-kb resolution Hi-C map of GM12878 (obtained by combining the two replicates), MUSTACHE resulted in 18,068 loop calls for *q* value < 0.05. For the 10-kb resolution GM12878 data, we obtained 14,045 loops from the combined contact map, and 11,872 and 10,976 loops from the primary and replicate experiments, respectively, using the same *q* value threshold of 0.05. For the combined 5-kb K562 Hi-C data from the same publication, we identified 8975 loops at a *q* value of 0.1. On the 5-kb resolution HFFc6 cell line, MUSTACHE called 16,132 loops from the Hi-C data and 36,494 loops from Micro-C data with the *q* value <0.01 (24,045 loops for *q* value <0.001). For the 1-kb resolution Micro-C data at *q* value threshold of 0.01, MUSTACHE reported 50,472 loops for HFFc6 and 11,231 loops for mESC data.

In order to assess the sensitivity of MUSTACHE to the number of reads used for sequencing Hi-C samples, we performed a downsampling analysis on the 5-kb resolution GM12878 Hi-C data with 3.7B intra-chromosomal valid reads. Our results on 2B, 1B, 900M, 800M, …, 100M-read downsampled maps showed that the true positive rate of MUSTACHE’s loops (compared to the full 3.7B-read map) was consistently high (over 80%) for all settings suggesting that a lower sequencing depth did not lead to false positive discoveries (Additional file 1: Figure S1). However, the recovery rate dropped to 66% and 51% when we used 2B or 1B reads, respectively. For 500M valid reads, MUSTACHE detected only one third of all loops reported on the full dataset (Additional file 1: Figure S1). Given that MUSTACHE detects two to three times the number of HiCCUPS loops to begin with, and also reports statistical significance, MUSTACHE will capture a substantial fraction of the strong loops on such Hi-C datasets. Regardless, to achieve high sensitivity in loop detection at 5 kb or higher resolution, we suggest to have at least one billion valid intra-chromosomal read pairs per Hi-C map.

### Comparison of loop calls from Mustache, SIP, and HiCCUPS on Hi-C data

**Fig. 2:**
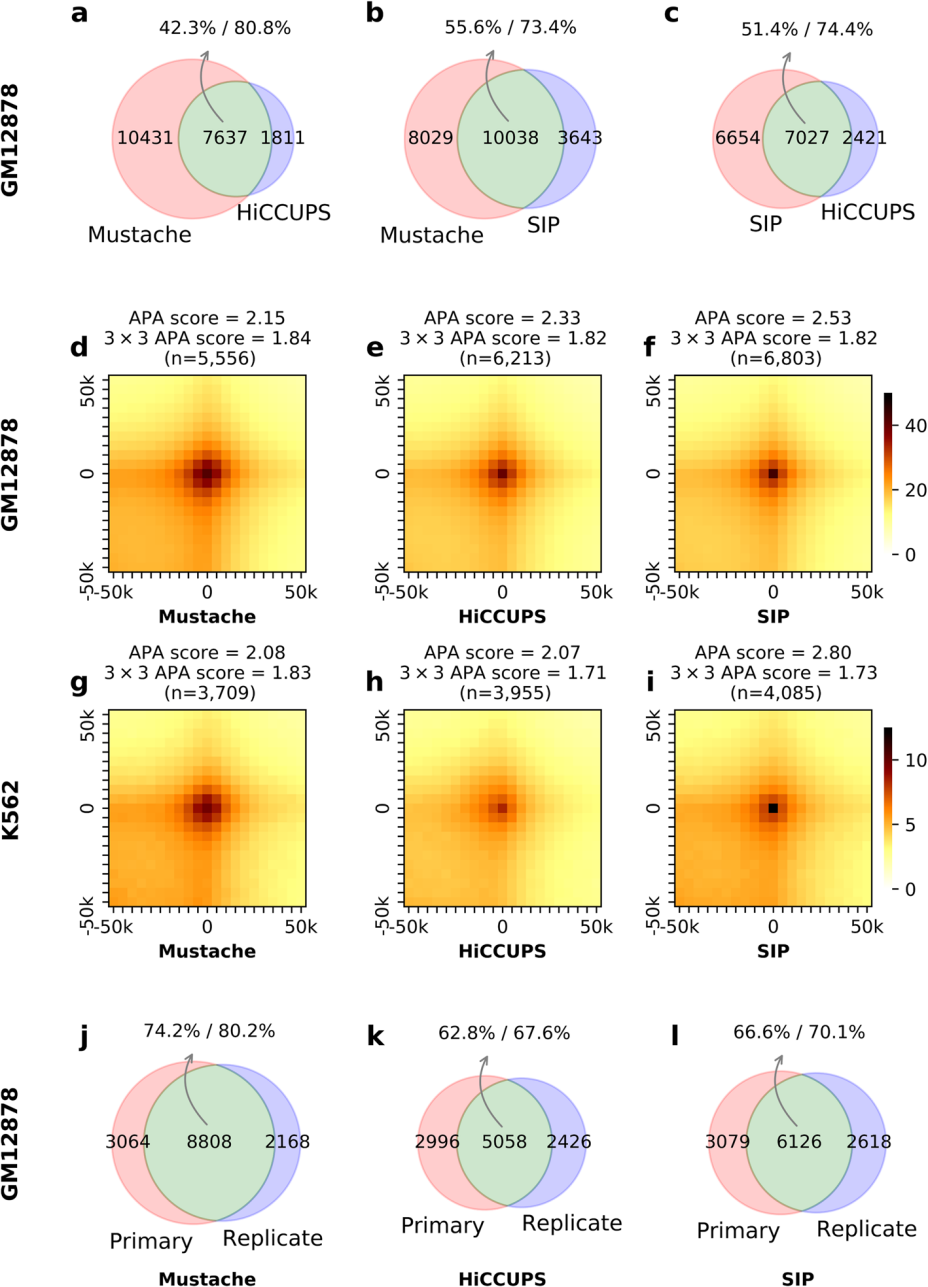
Comparison of loops detected by Mustache, HiCCUPS, and SIP from the GM12878 cell line Hi-C data. The agreement between **a** Mustache and HiCCUPS, **b** Mustache and SIP, and **c** SIP and HiCCUPS loops is shown as Venn diagrams for replicate-combined Hi-C contact maps. The overlap between the two loop sets is shown in green, and the percentages of overlap with respect to each set are reported separately. **d**–**i** APA plots for Mustache, HiCCUPS, and SIP loops in GM12878 and K562 cell lines. The APA score calculated with respect to the enrichment of (i) the center pixel and (ii) a 3×3 neighborhood in the center is reported above each plot. The overlap between reported loops on two replicates of GM12878 cell line is shown for **j** Mustache, **k** HiCCUPS, and **l** SIP. For Mustache and SIP, a *q* value threshold of 0.05 was used. For **j**–**l**, when replicates are analyzed separately, we use 10 kb resolution Hi-C contact maps

As discussed, methods that detect enrichment of contact counts with respect to a global background model tend to report a number of significant contacts a few orders of magnitude higher than loop callers with local filters such as HiCCUPS (∼ 10k) and MUSTACHE (∼ 20k) on the same data. Even though the global background models are valuable for finding potential interactions among functional elements such as enhancers and promoters, most of the detected enrichments do not correspond loop anchors demarcating domain boundaries and/or specific loops between convergent CTCF binding sites which are argued to occur due to loop extrusion [9, 14–16]. Therefore, we focused our comparative analysis with what we call here as “loop calling” methods, which are geared towards detecting the strongest and domain-demarcating pixels, such as the commonly used HiCCUPS method [9]. Using the publicly available Hi-C contact maps described above, we conducted several comparisons between MUSTACHE, HiCCUPS, and SIP loop calls. HiCCUPS loops were obtained directly from the GEO entry for that work [9]. First, we performed a genome-wide comparison of MUSTACHE results against 9448 and 13,681 GM12878 and 6057 and 8323 K562 loop calls from HiCCUPS and SIP, respectively. To compute the overlap between two methods, we defined two loop calls as *matched* if the ± 5-kb area around the center of one loop overlapped the other (inclusive of corners and edges). For this analysis, all loop calls by MUSTACHE and SIP were reported at 5 kb resolution, whereas HiCCUPS reported a mix of loops at 5 kb and 10 kb. Figure 2a–c illustrate that there was a good agreement among all three methods. MUSTACHE recovered nearly 81% and 73% of HiCCUPS and SIP loops in GM12878 (Fig. 2a, b) while SIP recovered 74% of HiCCUPS loops in the same cell line. When we compared loop calls from GM12878 to that of K562, we observed that MUSTACHE, HiCCUPS, and SIP reported a similar fraction of common loops between the two cell lines suggesting that the three methods have similar cell type specificity (Additional file 1: Figure S2).

Figure 2a–c show that MUSTACHE reported a substantial number of additional loops for GM12878 cell type compared to the other methods. In order to further evaluate this difference, we quantified how well each loop set was supported by the Hi-C data using aggregate peak analysis (APA) [9]. To generate APA plots, we aggregated contact counts over all detected HiCCUPS loops and top-*k*MUSTACHE and SIP loops (equal to that reported by HiCCUPS). Then, among these, we only kept the loops between 150 kb and 1 Mb distance range. The result is illustrated as a 21×21 heatmap (at 5 kb resolution) in which darker color indicates higher contact count. A strong dark pixel at the center of the heatmap indicates specific enrichment of Hi-C contacts for the loop calls with respect to their local background. The enrichment is also quantified by several APA scores, one of which is the ratio of the value of the center pixel to the average value of pixels 15–30 kb upstream and downstream [26]. Figure 2d–i show APA plots and APA scores on GM12878 and K562 cell lines for all reported HiCCUPS loops and top-*k*MUSTACHE and SIP loops (equal to that reported by HiCCUPS). Then, among these, we only kept the loops between 150 kb and 1 Mb distance range. These results show that loops from all three methods exhibited comparable enrichment with respect to their local background. MUSTACHE loops had a more diffused center enrichment and accordingly lower APA scores compared to both HiCCUPS and SIP (APA score; Fig. 2d–i), a feature we found to be related to the multi-scale nature of loops detected by MUSTACHE. To support our claim, we recalculated the APA scores using the average of a 3×3 neighborhood at the center instead of the single center pixel. The recalculated APA scores showed that MUSTACHE and SIP perform similarly (3×3 APA score; Fig. 2d–i). Our evaluation of method-specific loops also showed the common loops between a pair of methods had higher APA scores compared to loops that are specific to a single method. MUSTACHE-specific loops had lower APA scores compared to loops specific to either HiCCUPS or SIP; however, the enrichment pattern of MUSTACHE-specific loops closely mimicked that of loops common to multiple methods (Additional file 1: Figure S3).

To measure the agreement between MUSTACHE, HiCCUPS, and SIP loop calls from replicate experiments, we compared them on two replicates of GM12878 cell line. For MUSTACHE and SIP, we used a fixed *q* value threshold (0.05). Since HiCCUPS calls for individual replicates were at 10 kb resolution, MUSTACHE and SIP were also run on 10 kb resolution replicate-specific maps. For assessing whether two loops match each other, one from each replicate, we used the overlap of ± 5 kb area around the center of each loop as previously described. Figure 2j–l show that while all methods have a considerable overlap between replicates, MUSTACHE reported 3750 and 2682 more reproducible loops, for the fixed significance threshold of *q* value <0.05, compared to HiCCUPS and SIP, respectively. These results indicate that MUSTACHE did not compromise the reproducibility to obtain additional loops and had better self-consistency compared to SIP and HiCCUPS. The comparative analysis of the distribution of genomic distances also confirmed that all three methods lead to similar distance distributions for their loop calls (Additional file 1: Figure S4).

We also evaluated the performance of MUSTACHE and SIP in terms of runtime and memory utilization on two very high-depth Hi-C maps, namely GM12878 [9] and HFFc6 [12], both of which had over 4B valid read pairs. Both MUSTACHE and SIP were run on a single CPU (Intel(R) Xeon(R) Gold 5218 CPU, 2.30 GHz, 20 GB RAM limit) with one thread (Additional file 1: Table S2). The results indicate that both methods were very efficient and can run on personal computers for 5 kb resolution human or mouse contact maps without requiring specialized computing resources such as a compute cluster or GPUs as needed by HiCCUPS. For example, for the largest human chromosome (chromosome 1), MUSTACHE took 7–8 min and utilized 8–9 GB RAM to call loops on 5 kb resolution GM12878 and HFFc6 Hi-C maps. It is important to note that MUSTACHE’s memory consumption was mainly driven by reading the contact map, while the core of the algorithm utilized less than 1 GB RAM for each case.

### Mustache identifies additional loops with enrichment in expected features of chromatin looping

Here, we first evaluated the convergence of CTCF sites on the anchors of chromatin loops detected by MUSTACHE (“**M**”), HiCCUPS (“**H**”), and SIP (“**S**”) from the combined GM12878 Hi-C data at 5 kb resolution. When we considered the same number of loop calls from each method (top-*k*, *k*=9448), all three methods had similar number of loops where the two connected loci each contained a single CTCF binding motif (**M**, 2926; **H**, 2993; **S**, 2943), and among them, they had a similar fraction of cases where these motif pairs were in convergent orientation (**M**, 88.6*%*; **H**, 89.7*%*; **S**, 89.2*%*). When we used the fixed *q* value threshold of 0.05 (resulting in a total of 18,068 loops for MUSTACHE and 13,681 loops for SIP), we obtained **M** 5318 and **S** 4410 loops with a unique CTCF motifs on both ends, out of which **M** 83.1*%* and **S** 85.9*%* had convergent orientation. Overall, MUSTACHE detected 1734 additional convergent CTCF loops compared to HiCCUPS and 627 additional loops compared to SIP for GM12878, suggesting the existence of important structural loops that might have been missed by these other methods.

Next, we compared the enrichment of physical binding of structural proteins such as CTCF, RAD21, and SMC3 [26] on the anchors of detected loops for each method on the same Hi-C data (GM12878, 5 kb resolution). First, we determined the set of loci that were involved in at least one reported loop for each method. Then, for each such locus, we extended its length to 15 kb (similar to Rao et al. [9], see Figure 6C [9]) and checked whether it “overlaps” a ChIP-seq peak corresponding to a CTCF or cohesin (SMC3 and RAD21) binding site. When we compared the percentage of interacting loci overlapping ChIP-seq peaks according to the above criterion, we observed that for each ChIP-seq data, between 83 and 90% of interacting loci from each method overlap with the peak calls when equal numbers of loops are considered (top-*k* setting). On the full set of loop calls for MUSTACHE and SIP (*q* value threshold of 0.05), these percentages were **M** 76.8*%*, **S** 83.9*%* for CTCF; **M** 77%, **S** 84.4*%* for RAD21; and **M** 73.2*%*, **S** 81.2*%* for SMC3. These results suggest that interacting loci from all three methods have significant enrichment for overlapping peaks of known looping-related insulator proteins with more stringent methods showing higher enrichments.

We, then, performed a similar overlap analysis, but this time using promoter and enhancer annotations for the GM12878 and K562 cell lines as determined by ChromHMM [27]. More specifically, we counted the number of interacting pairs of loci, where one locus overlapped a promoter region, and the other locus overlapped an enhancer region. Here, we define the notion of overlap similar to ChIP-seq peaks discussed above, but instead using the ChromHMM annotation. The results showed that 40.9*%* (7389) of MUSTACHE loops in GM12878 (*q* value < 0.05) and 43.5*%* (3904) in K562 (*q* value < 0.1) connected a promoter to an enhancer (Additional file 1: Figure S5). The corresponding percentages for HiCCUPS and SIP were 39.6*%* (3741) and 37.4*%* (5116) of loops in GM12878 and 38.8*%* (2350) and 42% (3495) in K562, respectively. Similarly, 17.6*%* (3179) and 19.1*%* (1714) of MUSTACHE loops, 17.8*%* (1681) and 17.4*%* (1053) of HiCCUPS loops, and 15.6*%* (2134) and 18% (1498) of SIP loops connected a promoter to another promoter for GM12878 and K562 Hi-C data, respectively (Additional file 1: Figure S5). These results suggested that the proportion of loops overlapping regulatory elements were similar for MUSTACHE, SIP, and HiCCUPS with MUSTACHE reporting an overall higher number of such loops.

**Fig. 3:**
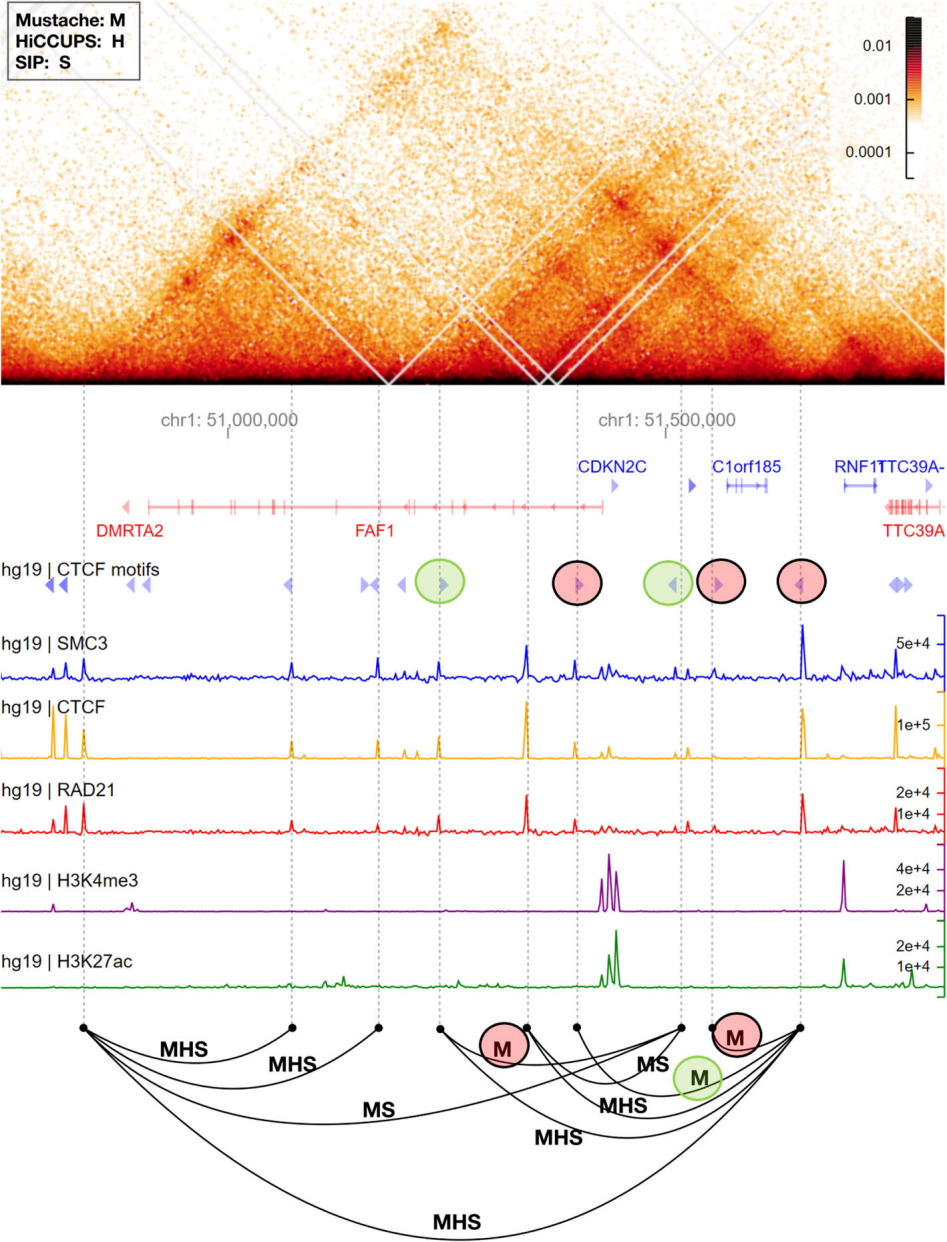
A comparison between Mustache, SIP, and HiCCUPS reported loop calls in a region of chromosome 1 for the GM12878 cell line (50.75–51.75 Mb). The Hi-C contact map is rotated 45^∘^ such that the main diagonal is horizontal (top). Below the contact map, we report genomic coordinates, gene annotations (genes on the negative strand are shown in red color), CTCF motifs and their orientation, and ChIP-seq signals for SMC3, CTCF, RAD21, H3K4me3, and H3K27ac (coverage tracks plotted by HiGlass). The bottom row demonstrates loop calls as arcs connecting two loci labeled by the initial letter of the methods by which they are detected (“M” for Mustache, “S” for SIP, and “H” for HiCCUPS)

**Fig. 4:**
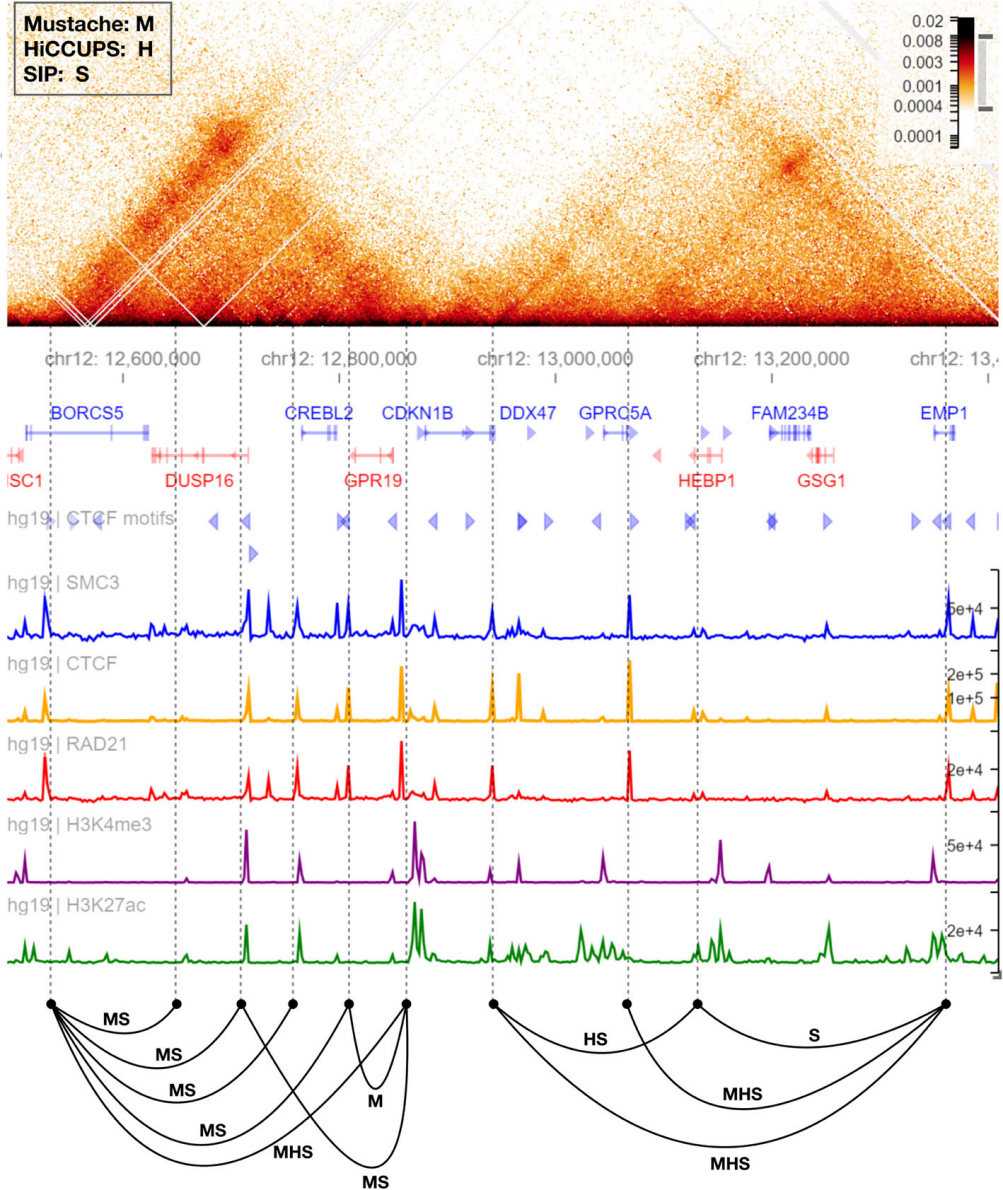
A comparison between Mustache, SIP, and HiCCUPS reported loop calls in a region of chromosome 1 for the GM12878 cell line (12.5–13.3 Mb). The display order is similar to that of Fig. 3

Lastly, we illustrate the relevance of MUSTACHE’s improved detection power on some selected regions for the GM12878 Hi-C data, using HiGlass for visualization [28]. Figures 3 and 4 provide a closer look at the regions 50.75–51.75 Mb on chromosome 1 and 12.5–13.4 Mb on chromosome 12, respectively. In these figures, we included the 45^∘^ rotated Hi-C heatmaps, together with gene annotations, CTCF motifs, and other relevant ChIP-seq signals. Loops are shown by black arcs labeled by the initial letter of the methods by which they are detected. Figure 3 shows that MUSTACHE detected all loops called by HiCCUPS and SIP for this locus and reported additional loops that connected ChIP-seq peaks of looping factors such as CTCF and cohesin binding. For example, three loops highlighted by red and green circles were detected only by MUSTACHE, and connected locus pairs that have CTCF and cohesin peaks and harbor convergent CTCF motifs. Figure 4 highlights another locus for which there were five loops that were detected by MUSTACHE and SIP, but not with HiCCUPS, each with cohesin and CTCF binding peaks on each end, and three of them with a convergent CTCF pairing. For this example, MUSTACHE missed two loops that were reported by either HiCCUPS or SIP and reported one loop missed by these two methods. Overall, these results highlight that MUSTACHE detects additional loops that are supported by the binding of looping-related factors, convergent CTCF motifs, and existence of regulatory elements such as enhancers and promoters, as well as with visual and quantifiable (APA plots) enrichments in the contact maps.

### Mustache recovers a larger fraction of cell type-matched HiChIP, ChIA-PET, and PCHi-C loop calls

**Fig. 5:**
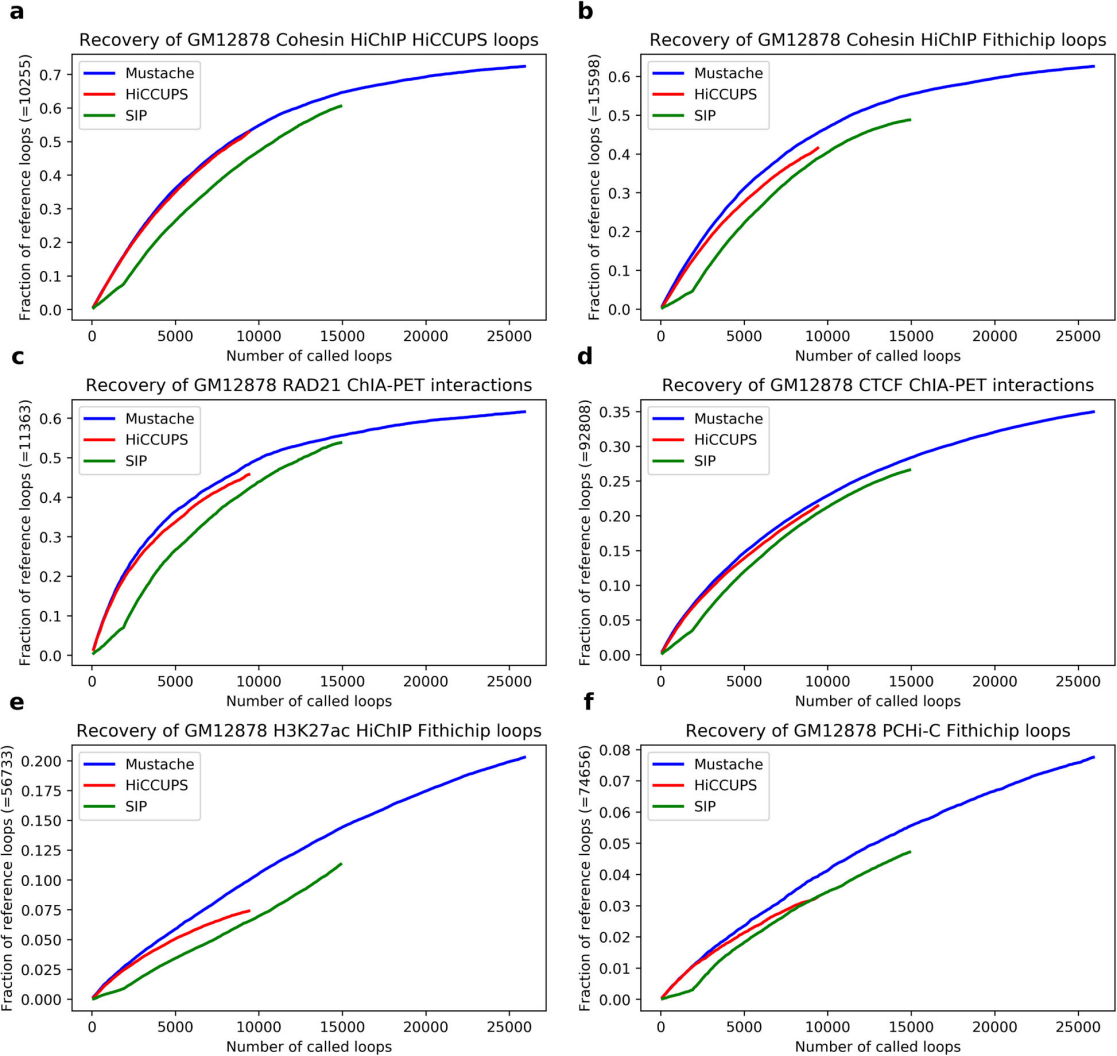
Comparison of the recovery of several reference loop sets by MUSTACHE, HiCCUPS, and SIP applied on the 5-kb resolution GM12878 Hi-C data. Recovery of **a** GM12878 cohesin HiChIP HiCCUPS loops, **b** GM12878 cohesin HiChIP FitHiChIP loops, **c** GM12878 RAD21 ChIA-PET interactions, **d** GM12878 CTCF ChIA-PET interactions, **e** GM12878 H3K27ac HiChIP FitHiChIP loops, and **f** GM12878 PCHi-C FitHiChIP loops

In the previous section, we showed that MUSTACHE detects additional loops (compared to HiCCUPS and SIP) that are supported by both genomic and epigenomic features. Here, we compared MUSTACHE, HiCCUPS, and SIP loop calls using published ChIA-PET, HiChIP, and PCHi-C loops as reference. In this experiment, we computed the number of ChIA-PET, HiChIP, and PCHi-C loop calls recovered by MUSTACHE, SIP, and HiCCUPS from the 5-kb resolution GM12878 data. Again, we used the matching criteria described in the reproducibility analysis to determine the overlap between two loop calls. Figure 5 a shows the recovery plot on GM12878 cohesin HiChIP data [29]. The *x*-axis represents the number of Hi-C loops called by MUSTACHE (blue), HiCCUPS (red), and SIP (green) sorted by their significance. We set HiCCUPS’ significance to be the median of the *q* values over the four local filters. MUSTACHE’s significance is the *q* value reported, as described in the “Methods” section. SIP’s significance is set to one minus the loop enrichment reported by the method. The *y*-axis represents the percentage of the HiCCUPS loops from cohesin HiChIP data that were recovered by each method. Figure 5 a shows that MUSTACHE and HiCCUPS on GM12878 Hi-C data had similar recovery patterns and both exceeded SIP’s recovery for equal number of loop calls from each method. However, MUSTACHE recovered more than 70% of all reference loops compared to less than 55% for HiCCUPS, even though the “reference” loops were previously determined using HiCCUPS on HiChIP data. We observed similar higher recovery for MUSTACHE when we used FitHiChIP [21] to call cohesin HiChIP loops (Fig. 5b). When we used ChIA-PET loops either from a RAD21 experiment (Fig. 5c) [30] or from a CTCF experiment (Fig. 5d) [31], MUSTACHE again provided a 10–15% improvement in recovery compared to HiCCUPS and and SIP. For H3K27ac HiChIP data [32] and for promoter capture Hi-C (PCHi-C) experiments [33] on the same cell line, the overall number of loop calls was several fold higher compared to each of the Hi-C methods we compared here due to different types of looping that these other experiments are designed to capture. However, MUSTACHE still provided an improved recovery when either the same number of loops (compared to HiCCUPS) or the whole set of calls were considered (Fig. 5e, f).

**Fig. 6:**
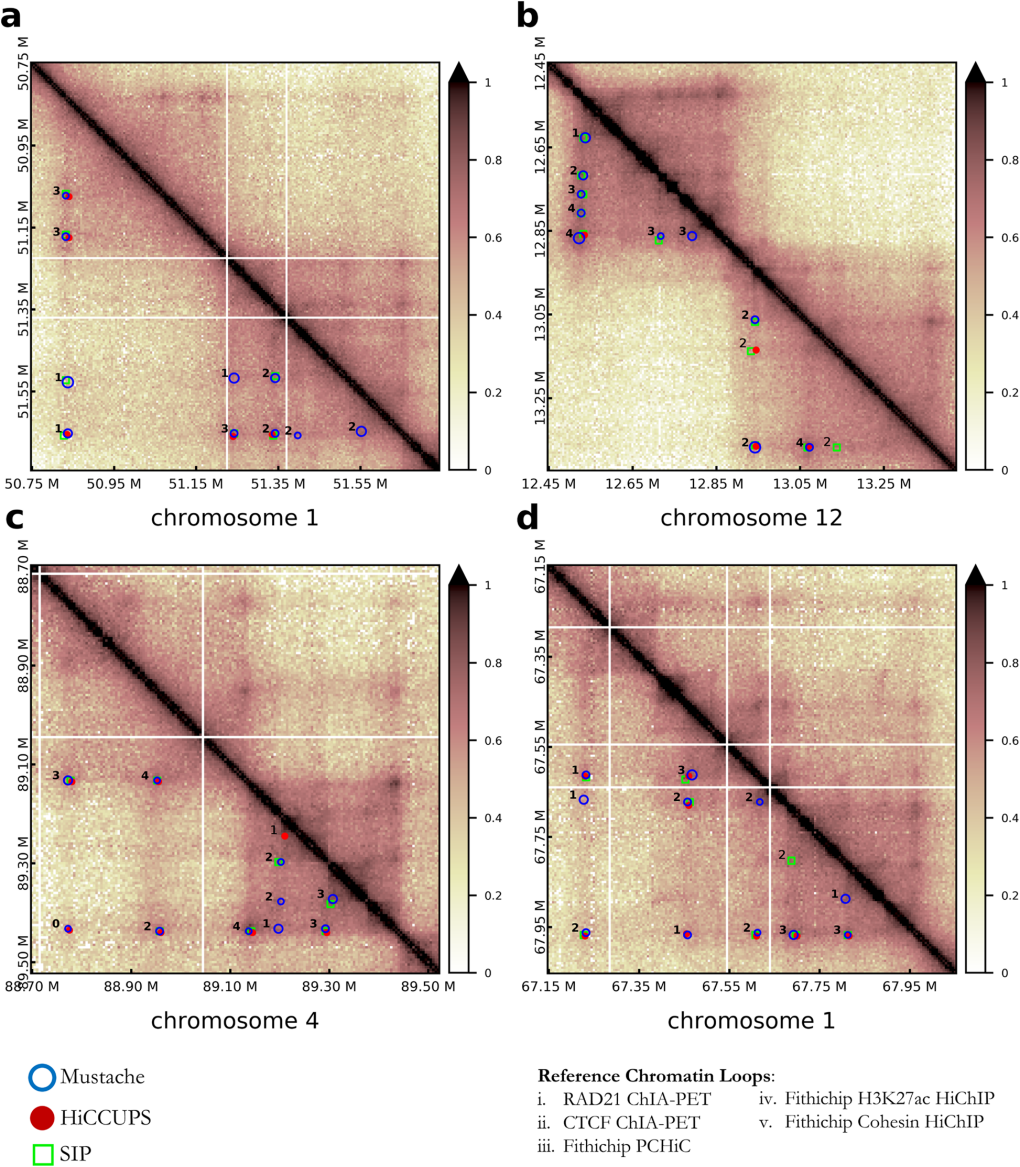
Four example regions showing Mustache, HiCCUPS, and SIP reported loops. Mustache, HiCCUPS, and SIP loops are represented by blue circles, red filled circles, and green squares on the lower diagonal matrices, respectively. The upper diagonal matrices were left untouched in order to allow visualization of contact patterns in the underlying Hi-C data. The loop calls of the three methods using GM12878 Hi-C data are shown for **a** 50.75–51.75 Mb region of chromosome 1, **b** 12.5–13.4 Mb region of chromosome 12, **c** 88.7–88.5 Mb region of chromosome 4, and **d** 67.2–68Mb region of chromosome 1

Taken together, the genome-wide analyses described above showed that MUSTACHE has better power in recovering loops identified from independent conformation capture experiments. To further elaborate on this point, we illustrate additional examples demonstrating that MUSTACHE-specific loops are supported by other conformation capture data. Specifically, for each Hi-C loop called by any of the three methods, we asked how many of the five reference loop sets listed in Fig. 6 support this Hi-C loop (number shown next to each circle), using the usual matching criteria defined above in the reproducibility analysis. The different radii of the circles illustrate the scale at which MUSTACHE detected these loops.

Figure 6a and b correspond to the two regions analyzed previously in Figs. 3 and 4, respectively. MUSTACHE (blue), HiCCUPS (red), and SIP (green) loops are highlighted on the lower diagonal, and the upper diagonal was left untouched to allow visualization of contact patterns. For the 1.2-Mb region on chromosome 1, Fig. 6a highlights six loop calls reported by all three methods, two reported by MUSTACHE and SIP, and three by MUSTACHE only. Each one of these three MUSTACHE-specific loops was supported at least by one reference set, and two were supported by two independent lines of evidence. Figure 6b shows that MUSTACHE and SIP both detected five chromatin loops lying on the border of a topologically associating domain [34], and potentially corresponding to a “stripe” region [35], out of which only one was detected by HiCCUPS (upper-left corner of the shown contact map). All four loops missed by HiCCUPS were supported by one or more reference sets. This same region also harbored one MUSTACHE-specific loop and one SIP-specific loop that were supported by three and two reference sets, respectively. For the last two regions, one on chromosome 4 (Fig. 6c) and one on chromosome 1 (Fig. 6d), we identified a total of five MUSTACHE-specific loops each of which was supported by at least one reference set. As expected, most of the detected loops fell inside or on the boundary of visible TADs for all methods. In general, there was high concordance between all three methods as indicated by 12 common loop calls in total. All these results taken together, along with the recovery analysis, suggest that the additional loops reported by MUSTACHE are not false positives, but they are likely *bona fide* looping events as they are supported by multiple lines of evidence and correspond to visibly and quantifiably enriched regions.

### Mustache detects loops that are consistent between Hi-C and Micro-C data

**Fig. 7:**
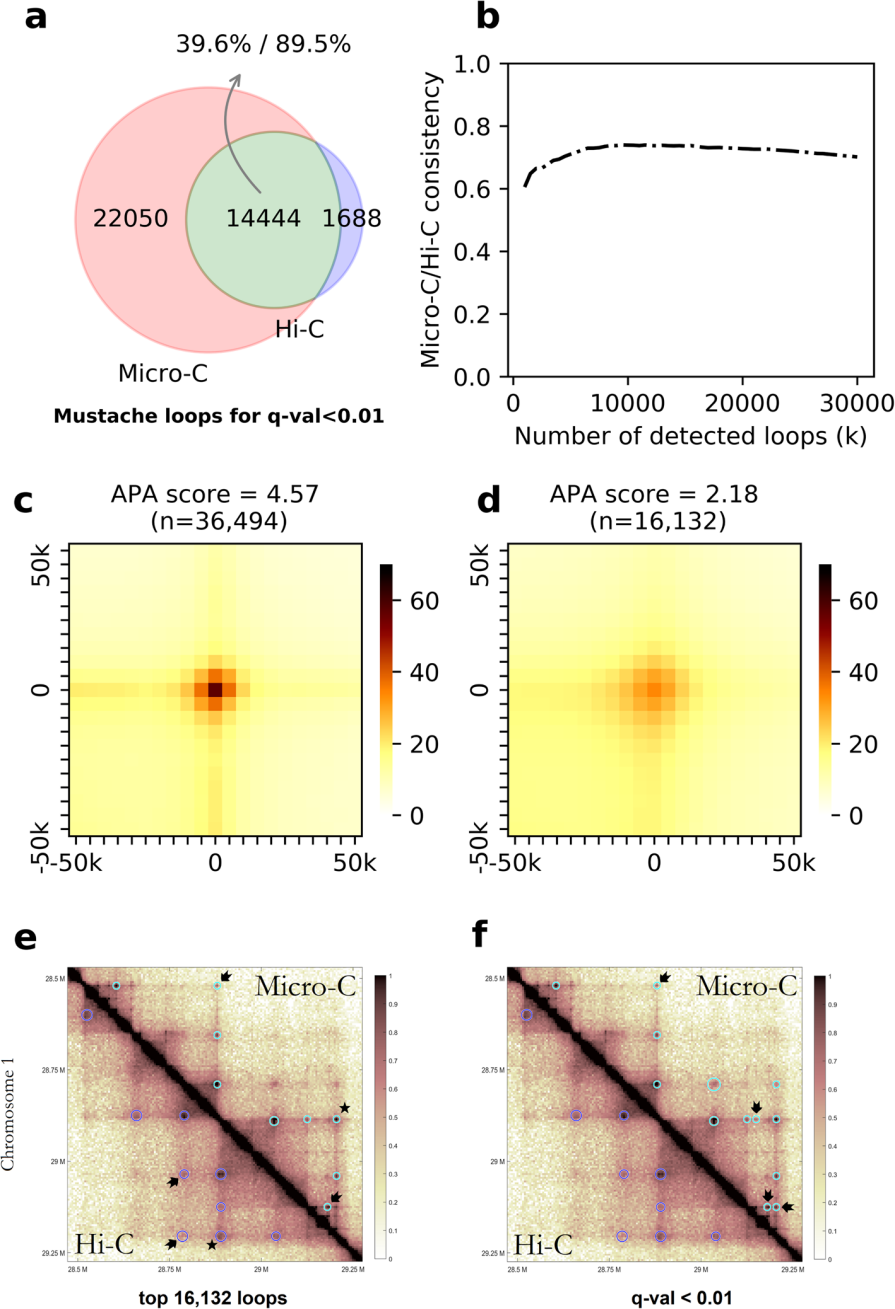
**a** The consistency between MUSTACHE loops detected using Micro-C and Hi-C data in HFFc6 cell line using a fixed *q* value threshold of 0.01, shown as a Venn diagram. **b** The consistency plot for MUSTACHE results between Hi-C and Micro-C for the top-*k* reported interactions for each contact map. The APA plots for MUSTACHE loops in HFFc6 cell line for **c** Hi-C and **d** Micro-C data. The APA score for the enrichment of center is reported above each plot. MUSTACHE reported loops in Hi-C (lower triangular) and Micro-C (upper triangular) of HFFc6 for **e** the top 16,132 significant interactions, and **f** a fixed *q* value threshold of 0.01. The loop call marked by a “ ⋆” was in common between Hi-C and Micro-C, but was detected at a smaller scale and has a stronger enrichment in Micro-C compared to Hi-C. The loops that are uniquely detected in either by Hi-C or by Micro-C are denoted by black arrows

The objective here was to assess the performance of MUSTACHE on Micro-C data and evaluate the consistency of Micro-C loops when compared to Hi-C loops on the same cell line. For this purpose, we used MUSTACHE on Hi-C and Micro-C data (using the same parameters) for the HFFc6 human cell line [12]. Figure 7a shows that MUSTACHE calls from Micro-C and Hi-C were largely consistent: 90% of Hi-C loops were also reported on the Micro-C data. However, MUSTACHE identified over 20k more loops from Micro-C, which is more than double the number of loops detected from Hi-C contact maps. We then assessed how this overlap changed if only considered the top-*k* most significant MUSTACHE loops from each dataset. Figure 7b shows the overlap fraction (*y*-axis) between the two datasets for different values of *k* ranging from 1k to 30k (*x*-axis). The overlap between Hi-C and Micro-C loops ranged between 60 and 74% with its maximum achieved when *k* is about 10k. This suggests that the ordering of loop candidates with respect to their significance is consistent between the two datasets, with Micro-C offering better statistical power for detection. In order to further understand this, we generated APA plots for loop calls from Hi-C and from Micro-C using the same contact maps for aggregation. Figure 7c and d show that the Micro-C loops had strikingly higher center pixel enrichment consistent with previous observations that Micro-C enriches signal-to-background ratio [12]. The reverse APA analysis (i.e., loop calls were taken from one dataset but the aggregation of contact patterns was computed using the other) showed that the Hi-C loops had a very striking center pixel enrichment in the Micro-C contact map (Additional file 1: Figure S6a), suggesting that MUSTACHE accurately detected the correct looping locations from Hi-C data. However, the local enrichment of loops detected from Micro-C was substantially lower when aggregate maps around the loop calls are created from Hi-C data compared (Additional file 1: Figure S6b) to the Micro-C data itself (Fig. 7d.)

Finally, we visualized Hi-C and Micro-C loop calls from MUSTACHE for an 800-kb region on chromosome 1. Figure 7e shows the loops called by MUSTACHE, when the tool was set to call the same number of loops on both datasets. Figure 7f shows loops called by MUSTACHE using a significance threshold of 0.01. We used a common normalized color scale for the contact map visualization to account for sequencing depth differences. Figure 7 e highlights a loop call (marked by a “ ⋆”) that was common between Hi-C and Micro-C, but was detected at a smaller scale and has a stronger enrichment in Micro-C compared to Hi-C. This example is consistent with the genome-wide patterns we summarized above using APA analysis. Also, in Fig. 7e, despite the fact that the same number of loops was called for both data types, MUSTACHE had different priorities in selecting loops due to the underlying differences between Hi-C and Micro-C outputs. This is also visible from the contact maps: loops that were uniquely detected in Hi-C or Micro-C are denoted by black arrows. When a fixed *q* value threshold is used, Fig. 7f shows that MUSTACHE called more loops from Micro-C compared with Hi-C including the two “Hi-C-specific” loops when top-*k* loops were considered (Fig. 7e). These additional Micro-C loops had strong local enrichment only visible on the Micro-C contact maps. This is possibly due to dilution of contact enrichment for some loops into multiple pixels for Hi-C in contrast to their concentration on a specific pixel when a higher resolution digestion system is used such as Micro-C. Taken together, all these experimental results show MUSTACHE provides highly consistent results between Micro-C and Hi-C data, but can leverage the unique strengths of each protocol to discover loops that are specifically enriched by that assay.

By taking advantage of the high-depth Micro-C experiments, we investigated loops beyond 5 kb resolution and included regions below the commonly used genomic distance threshold of 20 kb, both of which have been challenging to date even with deeply sequenced Hi-C datasets [9, 13]. For this experiment, we applied MUSTACHE on the 1-kb resolution Micro-C contact map of mouse embryonic stem cells (mESCs) [25] as well as human foreskin fibroblast cells (HFFc6) using a genomic distance range of 5 kb to 2 Mb. For these settings, MUSTACHE reported a total of 11,231 loops for mESC and 50,472 loops for HFFc6 cell lines both for *q* value <0.01. About one third of the reported loops had a genomic distance < 50 kb as apposed to only 4% for our 5 kb resolution analysis for both cell lines (Additional file 1: Figure S7). These included 1670 and 6823 loops for mESC and HFFc6, respectively, that are within 20 kb distance, a distance range generally ignored when studying 5 kb resolution contact maps. In this setting, MUSTACHE also identified loops that are > 1 Mb distance suggesting detection of both very short- and very long-range loops is possible from 1 kb data (Additional file 1: Figure S8). We then evaluated the support of MUSTACHE’s high-resolution loop calls by performing an APA analysis. The APA plots were generated by aggregating Micro-C contact counts in a ± 10-kb neighborhood (5 times shorter than 5 kb resolution) around the detected loops (Additional file 1: Figure S9) showing a significant enrichment of the loop calls compared to their local background (APA score of 4.79 for mESC and 6.71 for HFFc6). Next, we evaluated the enrichment of the structural marker CTCF [13] on the anchors of detected 1 kb loops for mESC Micro-C data. We found that 85% of the loop anchors (after extension to 5 kb rather than 15 kb as was used for 5 kb resolution analysis) overlap with CTCF binding, showing that the majority of detected loops in 1 kb resolution are associated with CTCF signal. We also computed the number of detected loops that involve promoters and enhancers (when loop anchors extended to 5 kb) using ChromHMM annotations showing that 3567 (32%) connect promoters to enhancers while 2148 (19%) connect promoters to promoters suggesting that a majority of these high-resolution calls may relate to gene regulation.

## Conclusions

The rapid adoption of Hi-C and its variants has fueled efforts in the development of computational tools to study 3D chromatin organization including chromosome compartments, topologically associating domains and chromatin loops [8, 9, 34, 36, 37]. Here, we presented a novel method for the identification of loops that uses a scale-space representation of Hi-C and Micro-C contact maps. Our novel multi-scale approach allows MUSTACHE to account for the dependencies among neighboring pixels (both spatially and across resolutions) of the contact map, which are ignored by methods that use global background estimates for significance calculation.

Our experimental results on Hi-C and Micro-C contact maps show that MUSTACHE robustly identifies looping events including those reported by HiCCUPS [9] and SIP, as well as those detected by independent chromatin conformation capture experiments, including ChIA-PET, HiChIP, and promoter capture Hi-C. MUSTACHE has several advantages, namely (i) it is scalable to kilobase-resolution human/mouse genome contact maps on standard laptop or desktop computers (few minutes per chromosome for 5 kb resolution human cell contact maps); (ii) it has better reproducibility of loop calls from replicate experiments; (iii) it provides higher statistical power that results in a higher number of chromatin loops with the additional loops strongly supported by genomic and epigenetic features; (iv) it produces highly consistent loop calls when comparing Hi-C and Micro-C data, allowing a robust comparative analysis of the two methods; and (v) it can handle Micro-C data for studying loops within genomic distances below 20 kb (at 1 kb resolution), an advance over most tools published to date. In summary, MUSTACHE represents a significant improvement over the state-of-the-art for the analysis of chromatin organization from high-resolution Hi-C and Micro-C contact maps.

## Methods

### Scale-space modeling

Objects in real world, as opposed to mathematically defined abstract entities such as points and lines, are composed of a variety of structures and textures at different scales which often makes them difficult to detect in the absence of a priori knowledge about their true scales. A way of addressing this challenge is to describe each object at multiple scales. In the specific problem on contact maps we are interested here, significant chromatin interactions are “blob-shaped objects” with a scale that depends on their size and other properties of the interacting genomic regions (e.g., CTCF binding, presence of regulatory elements).

Scale-space theory is a framework developed by the Computer Vision community for multi-scale representation of image data. In scale-space theory, each image is represented as a set of smoothed images. In order to build a scale-space representation of an image, a gradual smoothing process is conducted via a kernel of increasing width, producing a one-parameter (i.e., kernel size) family of images. This multi-scale representation makes it possible to detect smaller patterns at finer scales, while allowing the detection of larger patterns at coarser scales (Fig. 1a). The most common type of scale-space representation uses the Gaussian kernel because of its desirable mathematical properties. In particular, the causality property of Gaussian kernel guarantees that any feature at a coarse resolution scale is caused by existing features at finer resolution scales. This property makes sure that the smoothing process cannot introduce new extrema in the coarser scales of the scale-space representation of an image [38], which is critical for the problem we tackle here.

The Gaussian-kernel scale-space representation of an image *A*(*x*,*y*) is a function *L*(*x*,*y*,*σ*) obtained from the convolution of a variable-scale Gaussian *G*(*x*,*y*,*σ*) with the input image, as follows:

$$L(x,y,\sigma) = G(x,y,\sigma)*A(x,y), $$

where ∗ represents the convolution operation in *x* and *y*, and

$$G(x,y,\sigma) = \frac{1}{2\pi {\sigma}^{2}}e^{-\frac{ x^{2}+y^{2}}{2{\sigma}^{2}}} $$

is a 2D Gaussian (see [39] for more details).

Blob-shaped objects can be typically detected in an image by finding the strong responses in the application of the *Laplacian of the Gaussian* operator with an image, as follows:

$$\begin{array}{@{}rcl@{}} {\nabla}^{2} & = L_{xx} + L_{yy} \end{array} $$

Lindeberg showed that the normalization of the Laplacian with the factor *σ*^2^∇^2^ provides the scale invariance required for detecting blob-shaped objects at different scales [38]. According to Lowe [40], the scale-normalized Laplacian *σ*^2^∇^2^ can be accurately and efficiently estimated by the *difference-of-Gaussian* (DoG) function. Therefore, blob-shaped objects of varying scale can be detected from the scale-space maxima of the DoG function *D*(*x*,*y*,*σ*) convolved with the image, which can be computed from the difference of two nearby scales (in a scale-space representation) separated by a constant multiplicative factor *k*, as follows:

$$\begin{array}{@{}rcl@{}} D(x,y,\sigma) & = & (G(x,y,k\sigma)-G(x,y,\sigma))*A(x,y) \\ & = & L(x,y,k\sigma)-L(x,y,\sigma) \end{array} $$

### Development of MUSTACHE

Since a chromatin contact map can be represented by a digital image, we assume that a chromatin loop can be described as a somewhat-circular (blob-shaped) object with its own specific scale (that can be determined using the scale-space representation). Thus, MUSTACHE’s objective is to find blob-shaped regions of interactions with high statistical significance, i.e., regions with an average interaction significantly greater than expected. Due to random polymer interactions driven by one-dimensional genome proximity, interactions between pairs of loci that are closer in genomic distance are more frequent than interactions between loci at higher genomic distances. To account for the amplification of contact frequency due to 1D proximity, MUSTACHE performs a simple local *z*-normalization of the interaction frequencies in the contact map *A* with respect to their genomic distances along each diagonal *d*. Then, MUSTACHE re-scales the interactions by the logarithm of the expected interaction of the corresponding distance, as follows:

$$ \Tilde{A}(i,j) = \frac{A(i,j) - \mu_{d_{ij}}}{\sigma_{d_{ij}}} \log\left(1+\mu_{d}\right) $$

where *d*=|*j*−*i*|,*μ*_*d*_ is the average interaction on diagonal *d*, and $\mu _{d_{ij}}, \sigma _{d_{ij}}$ (not to be confused with Gaussian scale *σ*) are the local average and standard deviation along the diagonal *d* in a ± 1-Mb neighborhood, respectively. Then, MUSTACHE constructs the scale-space representation *D* of the normalized contact map *Ã*. As explained above, in order to compute *D*(*x*,*y*,*σ*), MUSTACHE convolves *Ã* with Gaussians that have increasing scales (i.e., *σ*,*kσ*,*k*^2^*σ*,⋯). This process produces a set of smoothed contact maps separated by a constant factor *k* in scale-space. MUSTACHE computes the difference of Gaussians (DoGs) by subtracting pairwise adjacent smoothed contact maps (Fig. 1a).

The scale-space representation comprises a set of “octaves,” each divided into *s* intervals, such that *k*=2^1/*s*^ [39]. In each octave, the scale starts with an initial value (*σ*_0_) which gradually increases by getting multiplied by the constant *k* until it is doubled. The next octave starts with the initial scale of 2×*σ*_0_, and this process continues until the whole representation is built. In this study, MUSTACHE uses two octaves of scale-space. MUSTACHE computes the *p* value $P_{\sigma _{k}}(x,y)$ for each pixel *D*(*x*,*y*,*σ*=*σ*_*k*_) by fitting a Laplace distribution on each scale of DoG, as follows:

$$ P_{\sigma_{k}}(x,y) = \mathbf{Pr}\left(X > D\left(x,y,\sigma=\sigma_{k}\right)\right) $$

where *X* is distributed according to the Laplace distribution. The choice of Laplace distribution was based on empirical observations. After computing the difference of Gaussians *D*(*x*,*y*,*σ*), MUSTACHE searches for local maxima in the 3D space over the parameters *x*, *y*, and *σ*. Specifically, MUSTACHE defines a 3D local maximum as a pixel (*x*,*y*,*s*_*i*_) (where *s*_*i*_ is a specific scale) that is greater than all its neighboring pixels at scale *s* (eight surrounding pixels) as well as its nine neighboring pixels in the scale above (*s*_*i*+1_) and nine neighboring pixels in the scale below (*s*_*i*−1_) within a 3×3×3 cube, as illustrated in Fig. 1b. Such local maxima are selected as candidate loops at scale *s*, and the rest are discarded (non-maxima suppression). In case a pixel is a local maximum at multiple non-adjacent scales, MUSTACHE reports the minimum *p* value across all scales for that specific pixel as its significance.

In order to find high-confidence and locally enriched loops, the set of detected candidates undergoes a few additional filtering steps. In the first step, MUSTACHE removes candidates that are not local maximum in at least two consecutive scales in a 3×3 two-dimensional neighborhood (i.e., it discards candidates that are local maximum at scale *s*_*i*_ but not in *s*_*i*−1_ and not in *s*_*i*+1_). Figure 1 b illustrates an example candidate pixel that passes the non-maxima suppression step (being a maximum in a 3×3×3 neighborhood) as well as the first filtering step. Observe that the pixel at (*x*,*y*) location (center pixel) is a local maximum in its 3×3 neighborhood at scales *s*_*i*_ and *s*_*i*+1_ but not a local maximum at scale *s*_*i*−1_. In the second filtering step, MUSTACHE finds connected components using 8-connectivity (i.e., a 3×3 neighborhood around each pixel) on a binary matrix in which an entry is set to one when that pixel is a candidate loop at any scale. For each connected component, MUSTACHE reports the single representative pixel that has the lowest *p* value. In the third filtering step, MUSTACHE filters out candidates that are located in sparse regions of the contact map. Specifically, it discards pixels whose neighborhood, as defined by a size equal to the *σ* of the scale the candidate was detected from, contains more than 20% pixels with zero count in the raw Hi-C contact map. Such pixels are likely enriched near repetitive and unmappable regions of the genome and may introduce false positives. In the fourth and final filtering step, candidates with contact count smaller than two times the expected count for that specific diagonal (i.e., the average contact count of all pairs with that corresponding genomic distance) are discarded. The remaining set of pixels are reported as loop calls together with their statistical significance and the scale of the Gaussian that the reported *p* value was identified from. MUSTACHE uses the Benjamini-Hochberg [41] procedure to correct *p* values for multiple hypothesis testing.

## Supplementary information

**Additional file 1** Integrated supplementary Figures and Tables. Contains figures from S1 to S9 and tables from S1 to S2.

**Additional file 2** Review history.

## Data Availability

A Python implementation of Mustache is freely available at https://github.com/ay-lab/mustacheunder the MIT license [42]. The version used in this article is available as a Zenodo archive with DOI 10.5281/zenodo.4046958[43]. This implementation works with commonly used file formats for Hi-C data including “.hic” and “.cool” formats. The data analyzed in this study are available on GEO, ArrayExpress, and 4DN data portal with the following accession numbers: GSE63525 [9], 4DNES2R6PUEK [12], 4DNESYTWHUH6 [12], GSE130275 [25], GSE80820 [29], GSE101498 [32], GSM1872886 [31], GSM1436265 [30], E-MTAB-2323 [33], GSE96107 [13], GSM733752 [44], GSM935376 [44], and GSM803416 [44]. These accession numbers are available in Additional file 1: Table S1 as well. All reported chromatin loops as well as the ChIP-seq data used in the manuscript are available for visualization through Washington University Epigenome browser [45]. To load the data in the Epigenome browser (http://epigenomegateway.wustl.edu), please download the JSON file of interest either from our GitHub page https://github.com/ay-lab/mustache/WashU-output[42] or from the Zenodo archive with DOI 10.5281/zenodo.4046958[43].
