# Supplementary material for: Mustache: multi-scale detection of chromatin loops from Hi-C and Micro-C maps using scale-space representation
Source: Genome Biol. 2020 Sep 30;21:256. doi: 10.1186/s13059-020-02167-0 (PMC7528378; doi:10.1186/s13059-020-02167-0)
Supplement: Supplementary file 1 — Additional file 1 Integrated supplementary Figures and Tables. Contains figures from S1 to S9 and tables from S1 to S2. [file 13059_2020_2167_MOESM1_ESM.pdf]

Mustache: Multi-scale Detection of Chromatin Loops from Hi-C and Micro-C Maps Using Scale-Space Representation

Additional file 1

Abbas Roayaei Ardakany, Halil Tuvan Gezer, Stefano Lonardi and Ferhat Ay

**Accession**	**Data Type**	**Cell Line**	**Factor**	**Reference**
GSE63525	Hi-C	GM12878	None	Rao et al. (2014) [9]
GSE63525	Hi-C	K562	None	Rao et al. (2014) [9]
4DNES2R6PUEK	Hi-C	HFFc6	None	Krietenstein et al. (2020)[5]
4DNESYTWHUH6	Micro-C	HFFc6	None	Krietenstein et al. (2020)[5]
GSE130275	Micro-C	mESC	None	Hsieh et al. (2020)[4]
GSE80820	HiChIP	GM12878	Cohesin (SMC1A)	Mumbach et al. (2016) [7]
GSE101498	HiChIP	GM12878	H3K27ac	Mumbach et al. (2017) [8]
GSM1872886	ChIA-PET	GM12878	CTCF	Tang et al. (2015) [10]
GSM1436265	ChIA-PET	GM12878	Cohesin (RAD21)	Heidari et al. (2014) [3]
E-MTAB-2323	PCHiC	GM12878	None	Mifsud et al. (2015) [6]
GSE96107	ChIP-seq	mESC	CTCF	Bonev et al. (2017) [1]
GSM733752	ChIP-seq	GM12878	CTCF	ENCODE [2]
GSM935376	ChIP-seq	GM12878	Cohesin (SMC3)	ENCODE [2]
GSM803416	ChIP-seq	GM12878	RAD21	ENCODE [2]

Table S1: Data sets used in this study

	GM12878		HFFc6	
	**Time (min)**	**Memory (GB)**	**Time (min)**	**Memory (GB)**
**Mustache**	78	9.2	70	8
**SIP**	152	1.8	169	1.7

Table S2: Runtime and peak memory usage of MUSTACHE and SIP on GM12878 and HFFc6 Hi-C data. Both methods were run on a single CPU (Intel(R) Xeon(R) Gold 5218 CPU - 2.30GHz - 20GB RAM limit) with one thread.

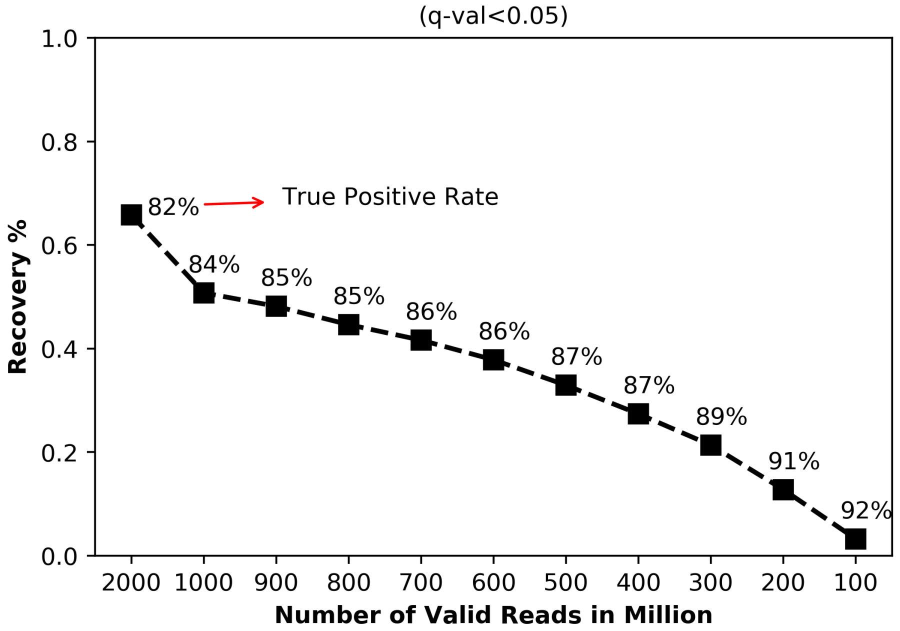

Figure S1: The effect of the sequencing depth on MUSTACHE's performance. The GM12878 cell line data with 3.7B valid intra-chromosomal reads was downsampled to 2B, 1B, 900M, 800M, ..., 100M reads to create downsampled contact maps (all at 5kb resolution). MUSTACHE was run on all these data sets using a q-value threshold of 0.05. The x-axis shows the downsampled contact map and the y-axis reports the percentage of the loop calls in the original 3.7B GM12878 map that was recovered by this downsampled map. The true positive rate is also reported for each dataset.

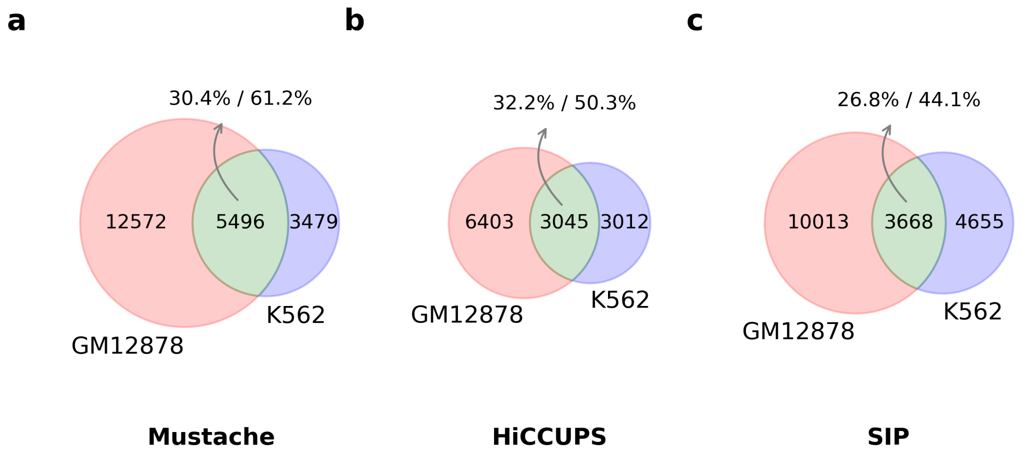

Figure S2: Reported chromatin loops in GM12878 and K562 cell lines and the overlap between them. The overlap is shown in green and the percentages of overlap with respect to each set are reported separately. **(a)** Mustache reported loops in GM12878 and K562. **(b)** HiCCUPS reported loops in GM12878 and K562. **(c)** SIP reported loops in GM12878 and K562. Mustache and SIP results were obtained using a q-value threshold of 0.05 for GM12878 and 0.1 for K562.

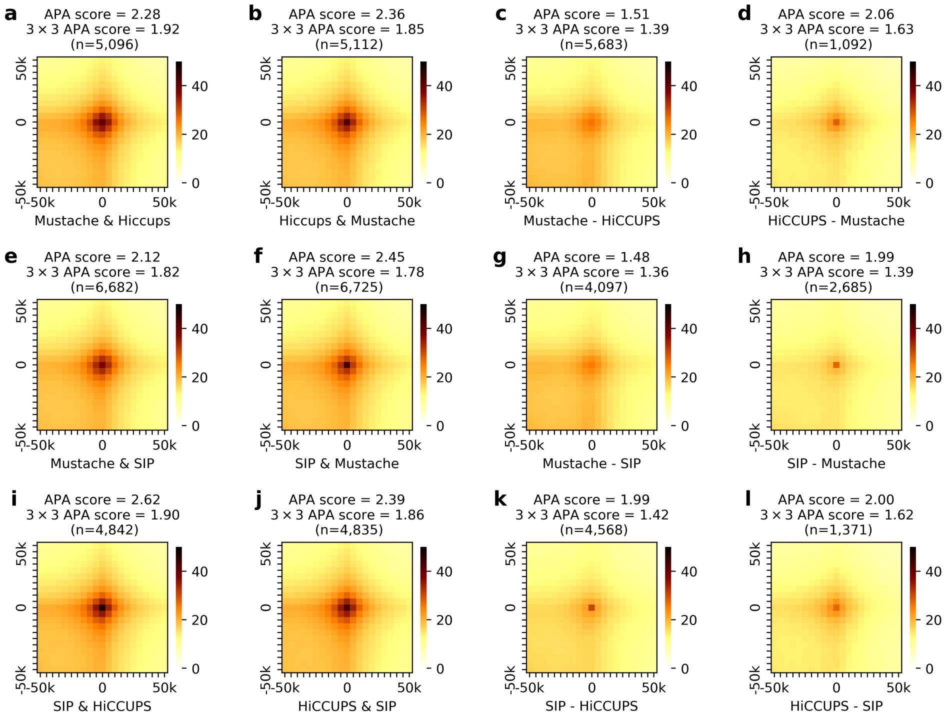

Figure S3: APA plots of shared loops between pairs methods (denoted by "&") and unique loops which are detected by one method and missed by the other (denoted by "-") on 5kb resolution Hi-C data for the GM12878 cell line. Mustache and SIP loops were called using a q-value threshold of 0.05. The APA plots are plotted for **(a)** shared loops between Mustache and HiCCUPS mapped on Mustache loop anchors, **(b)** shared loops between Mustache and HiCCUPS mapped on HiCCUPS loop anchors, **(c)** loops detected by Mustache and missed by HiCCUPS, **(d)** loops detected by HiCCUPS and missed by Mustache, **(e)** shared loops between Mustache and SIP mapped on Mustache loop anchors, **(f)** shared loops between Mustache and SIP mapped on SIP loop anchors, **(g)** loops detected by Mustache and missed by SIP, **(h)** loops detected by SIP and missed by Mustache, **(i)** shared loops between SIP and HiCCUPS mapped on SIP loop anchors, **(j)** shared loops between SIP and HiCCUPS mapped on HiCCUPS loop anchors, **(k)** loops detected by SIP and missed by HiCCUPS and **(l)** loops detected by HiCCUPS and missed by SIP.

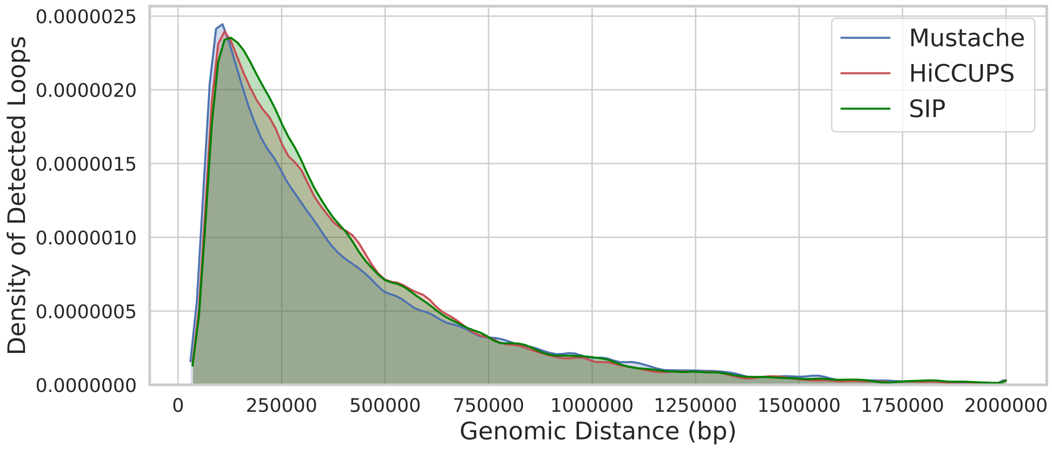

Figure S4: Comparison of the genomic distance distributions of loop calls reported by Mustache, HiCCUPS and SIP for the 5kb resolution Hi-C data from GM12878 cell line.

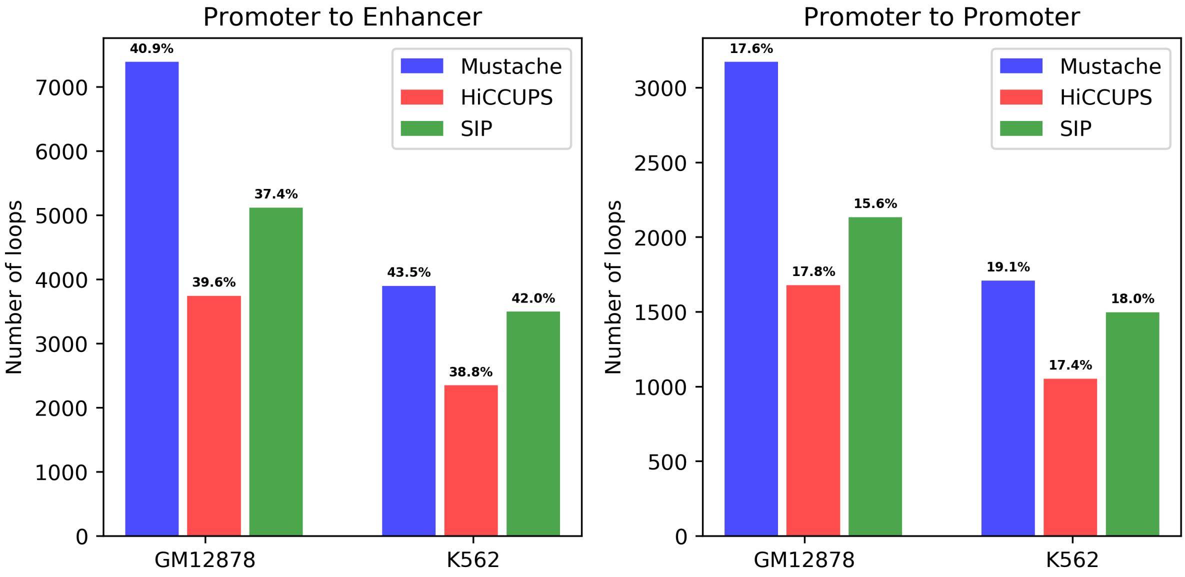

Figure S5: The number of loops detected by Mustache, HiCCUPS and SIP that connect promoters to enhancers (left) and promoters to promoters (right) according to ChromHMM chromatin state annotations. The percentage of such loops among all reported loops for each method is reported above each bar.

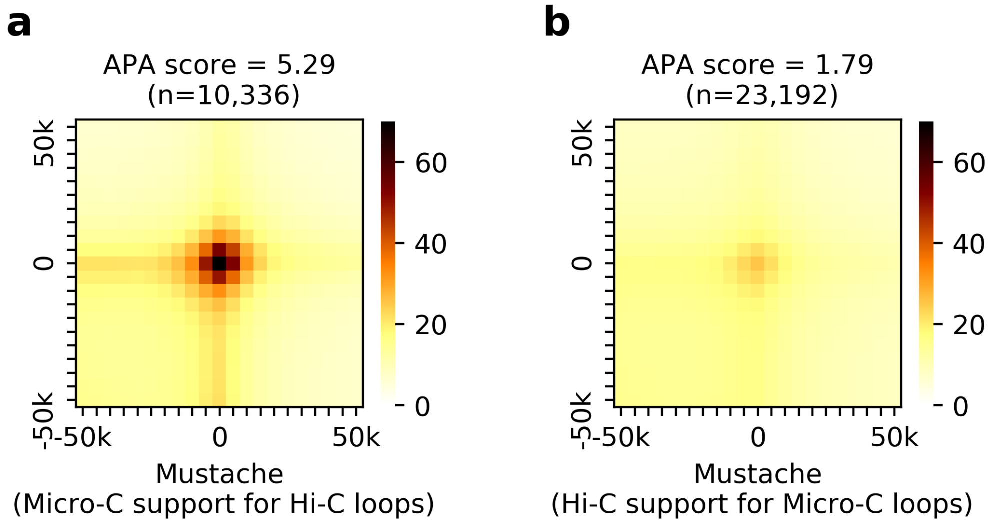

Figure S6: APA plots for Mustache's reported loops in HFFc6 cell line **(a)** detected using Hi-C data, supported by Micro-C contact maps, and **(b)** detected using Micro-C data, supported by Hi-C contact maps. Mustache loops were detected at a q-value threshold 0.01 for both datasets.

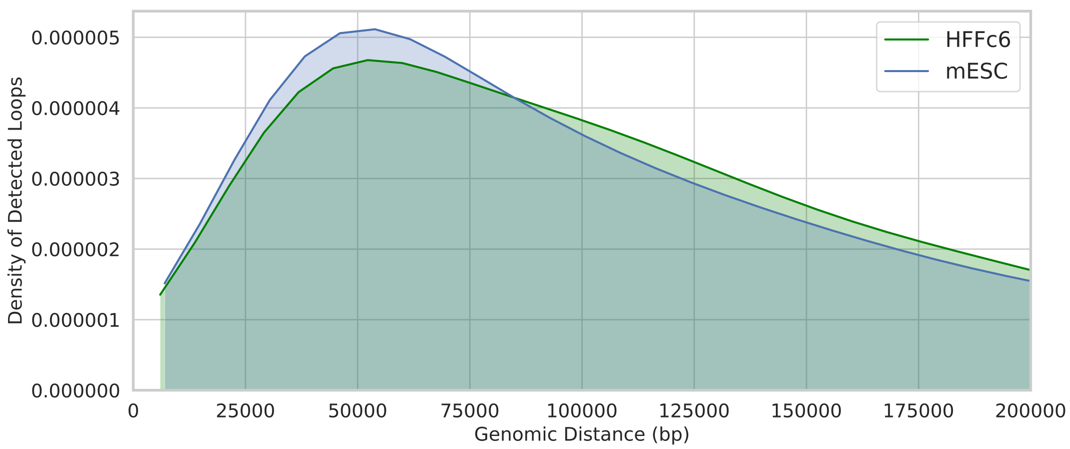

Figure S7: The genomic distance distribution of loop calls in the 5kb to 200kb range reported by Mustache on 1kb resolution HFFc6 and mESC Micro-C data.

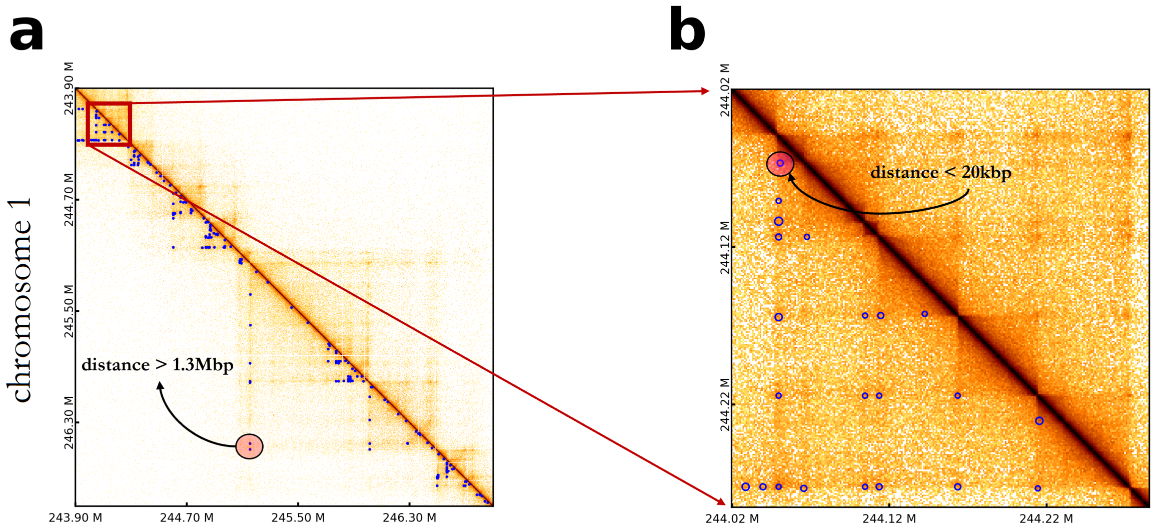

Figure S8: A region in chromosome 1 showing 1kb resolution loops detected by Mustache from HFFc6 Micro-C data. Observe that Mustache was able to detect loops at 1kb resolution in a wide range of genomic distances including: **(a)** >1Mb (3Mb region) and **(b)** <20kb (250kb region).

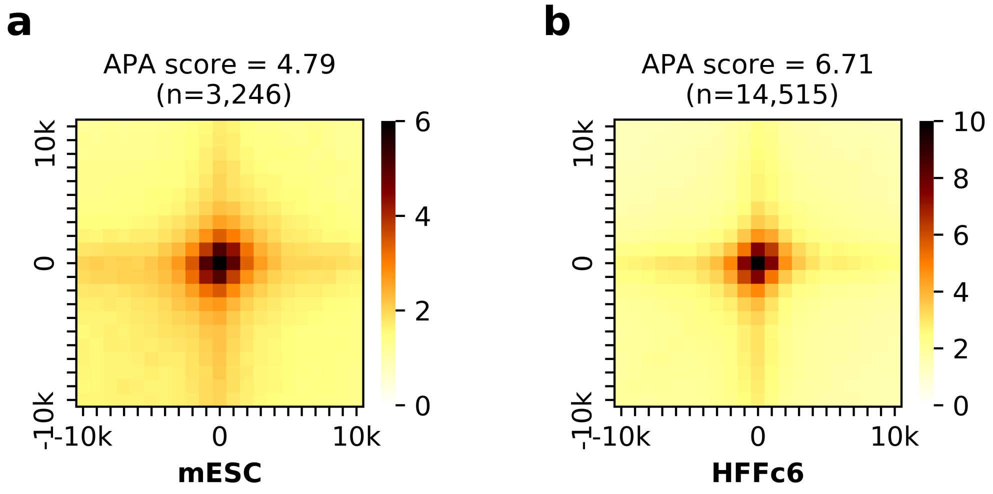

Figure S9: APA plots for detected loops in 1kb Micro-C data of **(a)** mouse embryonic stem cells (mESC) and **(b)** human foreskin fibroblast cells (HFFc6).

References

[1] Boyan Bonev, Netta Mendelson Cohen, Quentin Szabo, Lauriane Fritsch, Giorgio L Papadopoulos, Yaniv Lubling, Xiaole Xu, Xiaodan Lv, Jean-Philippe Hugnot, Amos Tanay, and Giacomo Cavalli. Multiscale 3D genome rewiring during mouse neural development. *Cell*, 171(3):557–572.e24, October 2017.

[2] ENCODE Project Consortium et al. An integrated encyclopedia of dna elements in the human genome. *Nature*, 489(7414):57, 2012.

[3] Nastaran Heidari, Douglas H Phanstiel, Chao He, Fabian Grubert, Fereshteh Jahanbani, Maya Kasowski, Michael Q Zhang, and Michael P Snyder. Genome-wide map of regulatory interactions in the human genome. *Genome Res.*, 24(12):1905–1917, December 2014.

[4] Tsung-Han S Hsieh, Claudia Cattoglio, Elena Slobodyanyuk, Anders S Hansen, Oliver J Rando, Robert Tjian, and Xavier Darzacq. Resolving the 3D landscape of Transcription-Linked mammalian chromatin folding, 2020.

[5] Nils Krietenstein, Sameer Abraham, Sergey V. Venev, Nezar Abdennur, Johan Gibcus, Tsung-Han S. Hsieh, Krishna Mohan Parsi, Liyan Yang, René Maehr, Leonid A. Mirny, Job Dekker, and Oliver J. Rando. Ultrastructural details of mammalian chromosome architecture. *Mol Cell.*, 78(3):554–565, 2020.

[6] Borbala Mifsud, Filipe Tavares-Cadete, Alice N Young, Robert Sugar, Stefan Schoenfelder, Lauren Ferreira, Steven W Wingett, Simon Andrews, William Grey, Philip A Ewels, et al. Mapping long-range promoter contacts in human cells with high-resolution capture hi-c. *Nature genetics*, 47(6):598, 2015.

[7] Maxwell R Mumbach, Adam J Rubin, Ryan A Flynn, Chao Dai, Paul A Khavari, William J Greenleaf, and Howard Y Chang. Hichip: efficient and sensitive analysis of protein-directed genome architecture. *Nature methods*, 13(11):919, 2016.

[8] Maxwell R Mumbach, Ansuman T Satpathy, Evan A Boyle, Chao Dai, Benjamin G Gowen, Seung Woo Cho, Michelle L Nguyen, Adam J Rubin, Jeffrey M Granja, Katelynn R Kazane, Yuning Wei, Trieu Nguyen, Peyton G Greenside, M Ryan Corces, Josh Tycko, Dimitre R Simeonov, Nabeela Suliman, Rui Li, Jin Xu, Ryan A Flynn, Anshul Kundaje, Paul A Khavari, Alexander Marson, Jacob E Corn, Thomas Quertermous, William J Greenleaf, and Howard Y Chang. Enhancer connectome in primary human cells identifies target genes of disease-associated DNA elements. *Nat. Genet.*, 49(11):1602–1612, November 2017.

[9] Suhas S P Rao, Miriam H Huntley, Neva C Durand, Elena K Stamenova, Ivan D Bochkov, James T Robinson, Adrian L Sanborn, Ido Machol, Arina D Omer, Eric S Lander, and Erez Lieberman Aiden. A 3D map of the human genome at kilobase resolution reveals principles of chromatin looping. *Cell*, 159(7):1665–1680, 18 December 2014.

[10] Zhonghui Tang, Oscar Junhong Luo, Xingwang Li, Meizhen Zheng, Jacqueline Jufen Zhu, Przemyslaw Szalaj, Pawel Trzaskoma, Adriana Magalska, Jakub Wlodarczyk, Blazej Ruszczycki, Paul Michalski, Emaly Piecuch, Ping Wang, Danjuan Wang, Simon Zhongyuan Tian, May Penrad-Mobayed, Laurent M Sachs, Xiaoan Ruan, Chia-Lin Wei, Edison T Liu, Grzegorz M Wilczynski, Dariusz Plewczynski, Guoliang Li, and Yijun Ruan. CTCF-Mediated human 3D genome architecture reveals chromatin topology for transcription. *Cell*, 163(7):1611–1627, December 2015.
