# Supplementary material for: Mustache: multi-scale detection of chromatin loops from Hi-C and Micro-C maps using scale-space representation
Source: Genome Biol. 2020 Sep 30;21:256. doi: 10.1186/s13059-020-02167-0 (PMC7528378; doi:10.1186/s13059-020-02167-0)
Supplement: Supplementary file 2 — Additional file 2 Review history. [file 13059_2020_2167_MOESM2_ESM.docx]

**1^st^ round**

**Reviewer 1**

The authors present Mustache, a method that detects contact peaks in Hi-C maps by applying concepts from scale-space theory in computer vision. The method involves smoothing the underlying map using Gaussian kernels with increasing standard deviation (scale) to reveal "blob"-like features. Taking differences of these smoothed maps approximates the Laplacian operator which has the nice property that local extrema emerge when the scale of the kernel matches the size of the blob. They apply local extrema detection in this "scale space" and some filtering criteria and thresholding to produce a list of Hi-C peak calls.

Overall, I think this work is an excellent fit of methodology to an application domain and Mustache is likely to be of broad utility to the Hi-C community. Scale-space theory provides a mathematical justification for the choice of convolution kernel for feature detection and offers the flexibility of searching across scales simultaneously, which is a strength over ad hoc kernels like those used by HiCCUPS. That said, I have a few questions for clarification and some suggestions that I believe would improve the presentation of the work.

1. Is there a particular rationale for the distance-normalization formula used? I understand using a locally defined z-score, but the need for the additional log rescaling wasn't obvious to me. Does the performance of Mustache change significantly if using the more classic observed/expected normalization or the locally-adjusted expected used by HiCCUPS?

2. The choice of the initial scale (sigma0) and subsequent ones. Is there a rationale for it? Could it be formulated in terms of a genomic length scale? That would be useful to guide users in applying Mustache to look for new blob features at different scales than CTCF-associated peaks. The terminology of "octaves" is also obscure to someone outside the computer vision field. My understanding from reading Lowe et al is that images are downsampled at each octave, but that doesn't seem to be what is done in Mustache. A clearer explanation about how the scale parameter is chosen and sampled geometrically would be helpful.

3. What is the rationale for fitting a biexponential distribution to the DoG maps for computing p-values? If it is simply based on an empirical observation, that is fine, but it is worth stating. Furthermore, have the authors explored whether an approach for significance testing similar to HiCCUPs's "lambda chunking" that groups candidate loops by their local expected can lower false positives?

4. Do the matchings of Mustache and HiCCUPS calls improve when using a broader area than 5kb from the peak center? Given that Mustache associates a "diameter" to each blob via the scale parameter (and the circular annotations in the figures suggest this too), would it make sense to use an area proportional to that scale? This relates to my question about associating a genomic length scale to a sigma value.

5. All figures with aggregate peak heatmaps need to use the same color value scale: the plots are simply not intelligibly comparable in their current state. If contrast is a problem, one could use a logarithmic scale that at least spans the same number of orders of magnitude in each plot. Another option that might help is to do the aggregate analysis on distance-normalized data instead of, or in addition to, the standard KR/ICE normalized data.

6. Figure 2: What I would like to see is APA plots of the **non-overlapping** peaks from each method. Perhaps a supplemental panel showing APA maps for the Mustache-only, Common and Hiccups-only peaks as given by the Venn diagrams in Fig2a,b. This would indicate how strong the peaks "missed" by either method tend to be and hint at specificity.

Minor comments:

1. The authors begin by defining the blob patterns they are searching for as **chromatin** loops and provide a biological description in terms of loop extrusion. For clarity of exposition I think it would be helpful to describe the appearance of the pattern to be detected, operationally define a term such as "loop" to refer to the pattern, but make it clear that a "chromatin loop" is an interpretation of the pattern. Indeed, many blob-like patterns in a Hi-C map are not related to loop-extrusion (and some are interchromosomal) and Mustache could be a valuable tool for these too!

2. Methods, paragraph 1: "Objects in [the] real world"

3. MAPQ <=30 should be >= 30

4. Figure 3: The caption mentions red arcs, but there aren't any.

5. Figure 6: If possible, place all loop annotations on one "triangle" of the matrix and leave the other bare, to make the calls easier to evaluate by eye.

6. Figure 7: Make the aspect ratio of the heatmaps in Fig 7e,f equal. State the number of loops obtained at qval < 0.01.

Finally, I do not think such an analysis is at all necessary for this publication, but might be a nice discussion point to highlight a potential strength of the flexibility of the Mustache approach: have the authors looked at common non-CTCF blobs that occur in many interesting Hi-C datasets? Some examples I can suggest are the punctate polycomb-polycomb interactions in mouse ES cells [Bonev et al, 2017 (https://doi.org/10.1016/j.cell.2017.09.043), McGlaughlin et al, 2019 (https://doi.org/10.1016/j.celrep.2019.10.031)], blobs connecting genes in olfactory neurons in trans [Mohanan et al, 2019 (https://doi.org/10.1038/s41586-018-0845-0)] and new peaks between hyperacetylated regions from [Rosencrance et al, 2020 (https://doi.org/10.1016/j.molcel.2020.03.018)]? Along the same lines, do the authors think their scale-space approach could be extended to detect loop extrusion-associated stripes?

**Reviewer 2**

Prediction of chromatin loops from chromatin-conformation-capture sequencing data is an important topic in the field of 4D Nucleome. The research group has developed a series of tools in this regards, including Fit-HiC for Hi-C, FitHiChIP for HiChIP, and now Mustache for this submission. In contrast to the Fit-HiC, which estimates a background model from global contacts, Mustache considers local contacts to estimate the background and does not specify a fixed-size of local neighborhoods. This is achieved by borrowing the idea of scale-space from computer vision to model chromatin loops from a contact map. Compared to the commonly used HiCCUPS, Mustache is highly time efficient and requires no GPU. It also detects many more reproducible chromatin loops than HiCCUPS and also more supported by ChIA-PET and HiChIP. Overall, Mustache is an important tool for the analyses of high resolution Hi-C or Micro-C contact maps, especially giving that more and more Hi-C libraries are being generated with billions of reads. Having said that, there are several important aspects that the authors will need to address:

1)	The authors compared Mustache only to HiCCUPS. There are two methods recently published: Cloops (Cao et al., 2020) and SIP (Rowley et al., 2020). As an article on "Method", the authors need to report a comparison of their method to the most recent developments on the same topic.

2)	Generating billions of reads for each sample is still far expensive for most laboratories. Actually, the majority of the published Hi-C data have only 500 million reads or less. What would be the minimal sequence depth for Mustache to make reasonable good predictions? Or how sensitive Mustache will be in regards to sequencing depth?

3)	The author reported that Mustache consumed only a few minutes to predict loops for each chromosome at a resolution of 5K with general-purpose CPUs. This is impressive. However, the author will need to document time efficiency, memory efficiency, CPU configuration, and number of threads for example in a Table when compare to alternatives.

4)	Mustache recovered many more loops anchored at H3K27ac-enriched regions than HiCCUPS (Fig 5e). As CTCF-anchored structural loops are generally depleted in H3K27ac, Mustache may recover more percentage of loops associated with promoters than HiCCUPS. Is this the case?

**Response to reviewers**

**Reviewer 1**

The authors present Mustache, a method that detects contact peaks in Hi-C maps by applying concepts from scale-space theory in computer vision. The method involves smoothing the underlying map using Gaussian kernels with increasing standard deviation (scale) to reveal "blob"-like features. Taking differences of these smoothed maps approximates the Laplacian operator which has the nice property that local extrema emerge when the scale of the kernel matches the size of the blob. They apply local extrema detection in this "scale space" and some filtering criteria and thresholding to produce a list of Hi-C peak calls. Overall, I think this work is an excellent fit of methodology to an application domain and Mustache is likely to be of broad utility to the Hi-C community. Scale-space theory provides a mathematical justification for the choice of convolution kernel for feature detection and offers the flexibility of searching across scales simultaneously, which is a strength over ad hoc kernels like those used by HiCCUPS. That said, I have a few questions for clarification and some suggestions that I believe would improve the presentation of the work.

We thank the reviewer for an extremely accurate summary and in-depth review of our work. We are also thankful for the positive evaluation and constructive comments, all of which we have addressed as described below.

1. Is there a particular rationale for the distance-normalization formula used? I understand using a locally defined z-score, but the need for the additional log rescaling wasn't obvious to me. Does the performance of Mustache change significantly if using the more classic observed/expected normalization or the locally-adjusted expected used by HiCCUPS?

The normalization that worked well for us was “local z-normalization” along the diagonals, where we only considered the non-zero pixels. Therefore, we included the global information (log of the global average at the corresponding distance) in the calculation to make sure that the normalized values do not change drastically due to the locality and sparsity differences.

We have tried Observed/Expected and the locally-adjusted expected normalizations used by HiCCUPS which, coupled with our method, led to the introduction of many obviously false positives and missing of true positive interactions. Therefore, we decided to continue to use our local z-normalization, which works well with our loop detection method.

2. The choice of the initial scale (sigma0) and subsequent ones. Is there a rationale for it? Could it be formulated in terms of a genomic length scale? That would be useful to guide users in applying Mustache to look for new blob features at different scales than CTCF-associated peaks. The terminology of "octaves" is also obscure to someone outside the computer vision field. My understanding from reading Lowe et al is that images are downsampled at each octave, but that doesn't seem to be what is done in Mustache. A clearer explanation about how the scale parameter is chosen and sampled geometrically would be helpful.

We thank the reviewer for bringing up these important points. In short, sigma is represented in terms of pixels, thus it can be formulated with respect to the genomic length and the type/size of the blob features we are looking for. For the current purpose addressed in this paper, i.e. the detection of blob-shaped loops at the appropriate resolutions for Hi-C, the default choice of sigma_0=1.6 with #octaves=2 covers all the potential blob sizes we were interested in. Our future work may include extending Mustache to other data types and potentially other features (e.g., stripes). We will update the description page on GitHub accordingly to make sure the users will have the necessary information to run Mustache on other data types and to discover other features accordingly.

Regarding the point about “octaves”, the necessary explanation from the paper is below:

We added: *“The scale-space representation comprises a set of “octaves”, each divided into s intervals, such that k= 2^1/s [39]. In each octave, the scale starts with an initial value (σ0) which gradually increases by getting multiplied by the constant k until it is doubled. Then the next octave starts with the initial scale of 2×σ0 and this process continues until the whole representation is built. In this study, Mustache uses two octaves of scale-space.”*

As it is stated in Lindeberg et al., the subsampling in each octave is solely done for computational purposes and it does not affect the detection results. For the purpose of this study we only needed two octaves (unlike image processing purposes where a large number of octaves is usually needed) and the method was already efficient enough, so we decided to not complicate the algorithm by adding the subsampling as the performance gain would not be significant.

3. What is the rationale for fitting a biexponential distribution to the DoG maps for computing p-values? If it is simply based on an empirical observation, that is fine, but it is worth stating. Furthermore, have the authors explored whether an approach for significance testing similar to HiCCUPs's "lambda chunking" that groups candidate loops by their local expected can lower false positives?

We thank the reviewer for the chance to clarify this. The choice of bi-exponential distribution was indeed based on our empirical observations. We have now clarified this in the text.

Regarding the Lambda-chunking:

Unlike HiCCUPS, the p-value we are calculating is not the enrichment of a pixel with respect to its neighborhood/s. Instead, we compute the probability of observing a circular region (weighted by a Gaussian filter of a particular scale) with a given DoG value in the contact map. Consequently, we were not able to employ the Poisson distribution.

However, we implemented a modified version of lambda-chunking by classifying our circular regions based on their background expected value of the center (calculated according to Rao et. al. 2014), and fitting a bi-exponential distribution to each class separately. The results for loops with distance < 2Mb was very similar to the previous approach (>90% match). However, the addition of this step significantly slowed down the method and increased the memory usage, because we had to calculate the local backgrounds and fit more models. On the other hand, the implementation of lambda-chunking enabled us to detect some loops with a genomic distance greater than 2Mb when applied to a broader range of contacts. After a careful consideration, we decided to not make this approach the “default” but to add it as an option to the method in case the user wants to discover loops from a broader range of genomic distances. We will continue to investigate whether this approach can be made efficient.

4. Do the matchings of Mustache and HiCCUPS calls improve when using a broader area than 5kb from the peak center? Given that Mustache associates a "diameter" to each blob via the scale parameter (and the circular annotations in the figures suggest this too), would it make sense to use an area proportional to that scale? This relates to my question about associating a genomic length scale to a sigma value.

We thank the reviewer for this suggestion. We made a careful choice of using a fixed size slack for matching to carry out a fair comparison with other methods. By increasing the slack from 5kb to 15kb, the recovery of HiCCUPS loops by Mustache in GM12878 only increases by a few percent (81% to 86%). Similarly, by using the scale (sigma) as the size of the slack the recovery increases to 86%. Therefore, we concluded that although the matching is associated with the scale (sigma) of the loops, a very large fraction of the loops can be already matched between methods/replicates using the stringent slack of 5kb.

5. All figures with aggregate peak heatmaps need to use the same color value scale: the plots are simply not intelligibly comparable in their current state. If contrast is a problem, one could use a logarithmic scale that at least spans the same number of orders of magnitude in each plot. Another option that might help is to do the aggregate analysis on distance-normalized data instead of, or in addition to, the standard KR/ICE normalized data.

We fixed the issue and used the same color scale for all the plots of the same dataset.

6. Figure 2: What I would like to see is APA plots of the **non-overlapping** peaks from each method. Perhaps a supplemental panel showing APA maps for the Mustache-only, Common and Hiccups-only peaks as given by the Venn diagrams in Fig2a,b. This would indicate how strong the peaks "missed" by either method tend to be and hint at specificity.

We have now included these results as Supplementary Figure 3. These results suggest that all method-specific loops have enrichments in the APA plots but to a lesser extent compared to the loops that are shared by two different methods (as expected). Similar to the general APA comparison, Mustache loops had lower APA scores compared to the two other methods mainly due to the multi-scale nature of our loop calls and how APAs are computed using the single center pixel. We have now included the discussion below to clarify these results:

We added: *“Figure 2d-i show APA plots and APA scores on GM12878 and K562 cell lines for all reported HiCCUPS loops and top-k Mustache and SIP loops (equal to that reported by HiCCUPS). Then, among these, we only kept the loops between 150kb to 1Mb distance range. These results show that loops from all three methods exhibited comparable enrichment with respect to their local background. Mustache loops had a more diffused center enrichment and accordingly lower APA scores compared to both HiCCUPS and SIP (APA score; Figure 2d-i), a feature we found to be related to the multi-scale nature of loops detected by Mustache. To support our claim, we recalculated the APA scores using the average of a 3×3 neighborhood at the center instead of the single center pixel. The recalculated APA scores showed that Mustache and SIP perform similarly (3×3 APA score; Figure 2d-i). Our evaluation of method-specific loops also showed the common loops between a pair of methods had higher APA scores compared to loops that are specific to a single method. Mustache-specific loops had lower APA scores compared to loops specific to either HiCCUPS or SIP, however, the enrichment pattern of Mustache-specific loops closely mimicked that of loops common to multiple methods (Supplementary Figure 3).”*

**Minor comments:**

7.1. The authors begin by defining the blob patterns they are searching for as **chromatin** loops and provide a biological description in terms of loop extrusion. For clarity of exposition I think it would be helpful to describe the appearance of the pattern to be detected, operationally define a term such as "loop" to refer to the pattern, but make it clear that a "chromatin loop" is an interpretation of the pattern. Indeed, many blob-like patterns in a Hi-C map are not related to loop-extrusion (and some are interchromosomal) and Mustache could be a valuable tool for these too!

We expanded our wording to include other potentially relevant patterns. Indeed, Mustache should be able to detect any blob-like pattern. In our response to 7.7 below we report on additional experimental results for some of the datasets suggested by this reviewer.

7.2. Methods, paragraph 1: "Objects in [the] real world"

7.3. MAPQ <=30 should be >= 30

7.4. Figure 3: The caption mentions red arcs, but there aren't any.

We have now fixed these three issues.

7.5. Figure 6: If possible, place all loop annotations on one "triangle" of the matrix and leave the other bare, to make the calls easier to evaluate by eye.

We have now fixed the comparison between methods (SIP is also included). For Hi-C vs Micro-C comparative plots, however, we believe this change would make things difficult to see as they would have to be drawn on two different heatmaps.

7.6. Figure 7: Make the aspect ratio of the heatmap.

We have now fixed this.

7.7. Finally, I do not think such an analysis is at all necessary for this publication, but might be a nice discussion point to highlight a potential strength of the flexibility of the Mustache approach: have the authors looked at common non-CTCF blobs that occur in many interesting Hi-C datasets? Some examples I can suggest are the punctate polycomb-polycomb interactions in mouse ES cells [Bonev et al, 2017 (<https://doi.org/10.1016/j.cell.2017.09.043>), McGlaughlin et al, 2019 (<https://doi.org/10.1016/j.celrep.2019.10.031)>], blobs connecting genes in olfactory neurons in trans [Mohanan et al, 2019 (<https://doi.org/10.1038/s41586-018-0845-0>)] and new peaks between hyperacetylated regions from [Rosencrance et al, 2020 (<https://doi.org/10.1016/j.molcel.2020.03.018)>]? Along the same lines, do the authors think their scale-space approach could be extended to detect loop extrusion-associated stripes?

These are all great suggestions and we have done our best to try Mustache on these different datasets/features during this round of revisions. We certainly intend to test and tune Mustache for other data types (e.g., ChIA-PET, HiChIP, PCHi-C), but as suggested, carrying out these additional experiments along with a thorough evaluation is beyond the scope of this paper.

Regarding suggestions about these recent datasets:

We first considered the data by McGlaughlin et al. (2019), however, as it is stated in their paper, their Hi-C experiments are not deep enough to call loops with high quality: “*since our own Hi-C data were not deep enough to call loops with high quality, we chose instead to take advantage of very deeply sequenced published data from mESCs (Bonev et al., 2017)*.” Therefore, we decided not to use this data given that Mustache performs best for high resolution data.

Next, we considered the dataset by Rosencrance et al. (2020) and the problem of identifying Megadomain-Megadomain interactions. As we show below, the pattern created by these interactions do not follow Mustache’s assumption about the shape of the feature to be detected.

However, we still applied Mustache on Hi-C data of TC-797 cells treated with DMSO in 100kb resolution and found 2,384 loops genome-wide which recovers only 44% of 289 reported intra-chromosomal Megadomain-Megadomain interactions. We should add that Mustache identifies many regions that are not reported in the paper which have patterns similar to Megadomain-Megadomain interactions (Figure below). Given that these patterns may also be related to compartment-level contact preferences or other contact map features, we did not feel we can make the claim that Mustache identifies Megadomain-Megadomain interactions with sufficient sensitivity and specificity.

**Reviewer 2**

Prediction of chromatin loops from chromatin-conformation-capture sequencing data is an important topic in the field of 4D Nucleome. The research group has developed a series of tools in this regard, including Fit-HiC for Hi-C, FitHiChIP for HiChIP, and now Mustache for this submission. In contrast to the Fit-HiC, which estimates a background model from global contacts, Mustache considers local contacts to estimate the background and does not specify a fixed-size of local neighborhoods. This is achieved by borrowing the idea of scale-space from computer vision to model chromatin loops from a contact map. Compared to the commonly used HiCCUPS, Mustache is highly time efficient and requires no GPU. It also detects many more reproducible chromatin loops than HiCCUPS and also more supported by ChIA-PET and HiChIP. Overall, Mustache is an important tool for the analyses of high resolution Hi-C or Micro-C contact maps, especially giving that more and more Hi-C libraries are being generated with billions of reads. Having said that, there are several important aspects that the authors will need to address:

We thank the reviewer for the positive evaluation of our work and their constructive comments, all of which we have addressed as described below.

1)	The authors compared Mustache only to HiCCUPS. There are two methods recently published: Cloops (Cao et al., 2020) and SIP (Rowley et al., 2020). As an article on "Method", the authors need to report a comparison of their method to the most recent developments on the same topic.

We thank the reviewer for the suggestions. We have now added the comparison with SIP even though this tool was not published (or available as a preprint) when we submitted our work to *Genome Biology* (late February 2020). Our comparisons suggest that, SIP is a significant improvement over HiCCUPS both in terms of loop detection sensitivity and scalability to high-resolution maps **(Figures 2** and **6**). However, Mustache outperformed SIP in several important evaluation criteria such as reproducibility between replicates and recovery of published loop calls from other data types **(Figures 2, 3, 5** and **6**). SIP, on the other hand, reported loops that resulted in better center pixel enrichment (APA-center score) compared to Mustache. We have shown that this was due to multi-scale nature of Mustache’s loop calling and APA scores were very similar when 3x3 center was considered instead of the single pixel (**Figure 2**). Also, for reporting Mustache APA scores, if we simply used the pixel with the most enriched count within 3x3 center instead of the center of the blob-like feature detected by Mustache, we would obtain higher APA scores compared to other methods. However, this pixel selection strategy reduced the reproducibility between replicates by ~10% and, hence, we chose not to make this our default option just for the sake of maximizing APA scores.

We added: “*While our work was under review, a method called Significant Interaction Peak caller (SIP) was published which also uses image processing techniques to identify chromatin loops in Hi-C data [19]. SIP applies a set of image processing operations (e.g., Gaussian blurring and contrast enhancement) to pre-process the contact maps and increase the contrast of the potential loops. Then SIP employs a local maxima detection algorithm to produce the preliminary list of candidate loops, which undergo several filtering steps to produce the final set of loop calls. Both HiCCUPS and SIP use a fixed representation of the contact map and a fixed-size local neighborhood to model the background intensities. Therefore, chromatin loops involving proximity of larger (or smaller) regions that lead to larger (or smaller) blobs in the contact map, as compared to the scale of the fixed representation, may not meet the local filtering criteria and will not be reported.”*

However, we decided to not include cLoops in the comparison due to several reasons as outlined in the main text (see below). Our preliminary analysis of cLoops calls downloaded directly from their GitHub page (<https://github.com/YaqiangCao/cLoops_supplementaryData>) for GM12878 cell lines suggested that 95% of the cLoops calls are not supported by any other method (HiCCUPS, SIP, Mustache) whereas we found 40-80% consistency for any pairwise comparison among HiCCUPS, SIP and Mustache.

We added: *“Another recent approach called cLoops is a local-enrichment method based on a modified DBSCAN clustering algorithm (cDBSCAN) that directly works with the paired-end tags/reads (PETs) to call loops [23]. cLoops uses a permuted lo-cal background to estimate statistical significance and can handle a broad range of chromatin conformation capture assays including Hi-C, ChIA-PET, HiChIP and Trac-loop. While cLoops is, to some extent, scale-free since it utilizes reads at their native resolution, cLoops is very inefficient both in terms of runtime (>1000x slower compared to SIP [19]) and memory use (>100GB per chromosome). Another down-side is that cLoops reports loops that are not supported by either HiCCUPS, SIP or Mustache, whereas these three methods have strong agreement among each other. For these reasons, we focused on comparing the three local enrichment-based methods, namely Mustache, SIP and HiCCUPS.”*

2)	Generating billions of reads for each sample is still far expensive for most laboratories. Actually, the majority of the published Hi-C data have only 500 million reads or less. What would be the minimal sequence depth for Mustache to make reasonable good predictions? Or how sensitive Mustache will be in regards to sequencing depth?

We evaluated the effect of the number of valid paired-end reads on loop calling of Mustache. We subsampled Rao et al. GM12878 cell line data (with combined replicates) with 3.7B intra-chromosomal reads to 2B, 1B, 900M, 800M,...,100M reads and ran Mustache for all datasets using the same FDR threshold (see below).

We added: *“In order to assess the sensitivity of Mustache to the number of reads used for sequencing Hi-C samples, we performed a downsampling analysis on the 5kb resolution GM12878 Hi-C data with 3.7B intra-chromosomal valid reads. Our results on 2B, 1B, 900M, 800M, . . . , 100M-read downsampled maps showed that the true positive rate of Mustache’s loops (compared to the full 3.7B-read map) was consistently high (over 80%) for all settings suggesting that a lower sequencing depth did not lead to false positive discoveries (Supplementary Figure 1). However, the recovery rate dropped to 66% and 51% when we used 2B or 1Breads, respectively. For 500M valid reads, Mustache detected only one third of all loops reported on the full dataset (Supplementary Figure 1). Given that Mustache detects two to three times the number of HiCCUPS loops to begin with, and also reports statistical significance, Mustache will capture a substantial fraction of the strong loops on such Hi-C datasets. Regardless, to achieve high sensitivity in loop detection at 5kb or higher resolution, we suggest to have at least one billion valid intra-chromosomal read pairs per Hi-C map.”*

3)	The author reported that Mustache consumed only a few minutes to predict loops for each chromosome at a resolution of 5K with general-purpose CPUs. This is impressive. However, the author will need to document time efficiency, memory efficiency, CPU configuration, and number of threads for example in a Table when compared to alternatives.

We thank the reviewer for this suggestion. We have now compared the runtime and memory use (as well as run configurations) of Mustache and SIP on two very high-depth datasets (Supplementary Table 2).

We added: *“We also evaluated the performance of Mustache and SIP in terms of runtime and memory utilization on two very high-depth Hi-C maps, namely GM12878 [9] andHFFc6 [12], both of which had over 4B valid read pairs. Both Mustache and SIP were run on a single CPU (Intel(R) Xeon(R) Gold 5218 CPU - 2.30GHz - 20GBRAM limit) with one thread (Supplementary Table 2). The results indicate that both methods were very efficient and can run on personal computers for 5kb resolution human or mouse contact maps without requiring specialized computing resources such as a compute cluster or GPUs as needed by HiCCUPS. For example, for the largest human chromosome (chromosome 1), Mustache took 7-8 minutes and utilized 8-9GB RAM to call loops on 5kb resolution GM12878 and HFFc6 Hi-C maps. It is important to note that Mustache’s memory consumption was mainly driven by reading the contact map, while the core of the algorithm utilized less than 1GB RAM for each case.”*

As HiCCUPS requires GPUs and since we used HiCCUPS loop calls directly from their published GEO archive, we did not report these measures for HiCCUPS. A detailed report of runtimes for HiCCUPS, cLoops and SIP can be found in the SIP paper (Rowley et al. 2020), which is consistent with our report here.

4)	Mustache recovered many more loops anchored at H3K27ac-enriched regions than HiCCUPS (Fig 5e). As CTCF-anchored structural loops are generally depleted in H3K27ac, Mustache may recover more percentage of loops associated with promoters than HiCCUPS. Is this the case?

We thank the reviewer for this great suggestion. Indeed, Mustache reports more loops compared to both HiCCUPS and SIP that are associated with promoters (either between two promoters (P-P) or between an enhancer and a promoter (E-P)) as shown in Supplementary Figure 4 (see also below).

Also, Mustache reported more loops with at least one anchor associated with a promoter regardless of the second anchor. For example, on the GM12878 cell line Mustache, HiCCUPS and SIP reported 9946, 5224 and 7189 loops associated with promoters, respectively, with the same matching criteria discussed in the paper.

**2^nd^ round**

**Reviewer 1**

All of my questions and suggestions have been satisfactorily addressed.

Only one very minor aesthetic comment: the heatmaps in Fig7e,f still have unequal aspect ratio -- they would look much better if it were fixed to 1:1.

**Reviewer 2**

The authors have carefully and satisfactorily addressed all of my concerns.
